# Clinical Review, and Hospital Reports

**Published:** 1839-01-01

**Authors:** 


### Clinical Review.

### GUY'S HOSPITAL.

Guy's Hospital Reports. No. VII. October, 1838. Edited by *George H. Barlow*, M.A. &c. &c., and *James P. Babington*, M.A. &c. &c. 8vo. pp. 476. Six Plates. Highley, London, 1838.

The active officers of Guy's Hospital continue the experiment of publishing periodical Reports, and of endeavouring to fill an annual volume with their own papers and their own cases. They deserve the highest credit for their energy and zeal.

The present number contains the following articles :—

A Contribution to the Pathology of Congenital Deafness; by Edward Cock. On the Effect produced upon the Pulse by Change of Posture; by William Augustus Guy, M.B. Cantab. Observations on Intus-susception, as it occurs in Infants; by John Gorham. Cases and Observations in Medical Jurisprudence; by Alfred S. Taylor. Some Observations on the Causes of Strangulation in Hernia, and on the Causes of Death: and also on the Rules of Treatment which such considerations enforce; by T. Wilkinson King. Chemical Examination of the Liquor Amnii; by G. O. Rees, M.D. F.G.S. &c. On Diabetic Blood; by G. O. Rees, M.D. F.G.S. &c. Observations on Abdominal Tumors and Intumescence; illustrated by Cases of Disease of the Spleen: with Remarks on the General Pathology of that Viscus; by Dr. Bright. Physiological Observations on the Muscles of the Eye; by Bransby B. Cooper, F.R.S.

We shall not take the articles exactly in their order.

### I. A Contribution to the Pathology of Congenital Deafness. By Edward Cock.

Mr. Cock starts with regretting, as many have done before him, that the ear still remains so much in the hands of quacks. The object of his communication is to help in taking it out of them. Passing over some observations on the little that is actually known of the causes of congenital deafness, we may allow Mr. Cock to state, that—

"Through the kindness of Mr. Watson and the Medical Officers of the Asylum for Deaf-and-Dumb, I have been enabled to inspect the bodies and examine the temporal bones of most of the children who have died in that institution during the last six years. The facts elicited from the dissection of the first six cases have already appeared in the Nineteenth Volume of the Transactions of the Royal Medical and Chirurgical Society. In three of these cases, I discovered in the organs of hearing such palpable deviations from the normal structure, as served to convince me that a congenital malformation does exist much oftener than has generally been supposed; and that a careful investigation of the auditory apparatus may, in many instances, throw light upon the pathology of deafness from birth. Since the publication of the Paper, several subsequent examinations have afforded some still more curious results, which may possibly open a wider field for speculation to the physiologist, as well as to the morbid anatomist." 291.

We may observe that out of six subjects examined, four presented lesions strictly congenital, and therefore bearing on the congenital deafness. In two instances, there was a partial deficiency of the semicircular canals. The extremities of these tubes opening into the vestibule were perfect; but the central portions were impervious, or, rather, did not exist at all. In the first case, the vertical and oblique semicircular canals were both impervious at their central portions. In this case only one ear could be examined. In the second case, both ears were examined. On the right side, the middle portions of the oblique and vertical canals were wanting, the bone presenting an appearance like that already described. On the left side, the horizontal and vertical canals exhibited a similar imperfection. The scala tympani, likewise, was terminated, at its larger extremity, by a bony septum, which separated it from the tympanum, and occupied the situation of the membrane of the fenestra rotunda.

In the third case, not a vestige was to be found of the fenestra rotunda on either side; the usual situation of the membrane being occupied by solid bone. The temporal bones of this child were exceedingly large, although soft and spongy in texture. The cavities were more than usually capacious; and the Eustachian tubes presented a remarkable development, being three or four times larger than common. On one side, the aqueduct of the vestibule readily allowed the passage of a large bristle: on the other side, the canal could not be traced through the bone, although its two extremities were more than usually expanded.

And, in the fourth case, the aqueduct of the vestibule was large enough to admit the passage of a small probe.

Such are the main circumstances connected with the six cases related in Mr. Cock's former communication.

Since 1835, he has obtained from the Deaf and Dumb Asylum, the opportunities for seven additional post-mortem examinations.

CASE 1.—A boy died with fever and acute cerebral symptoms, when twelve years old.

*Left Temporal Bone.*—The tympanum was completely filled by spongy and highly-vascular granulations, which appeared to grow from the mucous membrane lining the cavity; and to derive their vessels, which were numerous, large, and injected with blood, from the same source. This adventitious growth also occupied the mastoid cells, extended into the Eustachian tube, and adhered closely to the membrana tympani and the chain of small bones: it had no malignant character. The petrous bone was remarkably ill-shaped, very narrow from before to behind; the jugular fossa exceedingly large and excavated, so as to encroach upon the tympanum and Fallopian canal; the latter of which was laid open towards the fossa, when the periosteum was removed. The groove for the superior petrosal sinus was very broad and deep: in fact, the petrous portion was so scanty in its dimensions, and presented so little solid bone, that it was evident, on viewing its exterior, that the semicircular canals could not exist in their perfect state. The meatus auditorius internus was represented by a narrow slit; and the portio mollis became pulpy and transparent, as it descended into the canal. On cutting open the bone, the labyrinth presented the following appearances. The auditory canal, instead of being terminated by the cribriform plate forming the base of the modiolus, opened at once into a cavity of a somewhat conical shape; communicating with the vestibule by a very large aperture; and also with the tympanum, by means of the fenestra rotunda; through which last, the vascular granulations already described had found their way. This cavity, in fact, represented the mere external shell of a cochlea, but without a vestige of modiolus, spiral lamina, or scalæ: the auditory nerve entered it, and apparently expanded on its walls. The vestibule, or what answered to it in situation, was rendered irregular; on the one hand, by forming a continuous cavity with the imperfect cochlea; and on the other, by extending itself outwards so as to include that portion of bone usually embraced within the concavity of the horizontal semicircular canal. Not a trace, however, existed of either the horizontal or the oblique canal: the anterior opening of the vertical canal alone was present; but the canal itself suddenly stopped, after having completed about half its natural course. There was no aquæductus vestibuli.

*Right Temporal Bone.*—The tympanum was occupied by an adventitious growth, precisely similar to that on the left side. The petrous bone was more regular in its external configuration. The aquæductus vestibuli consisted of a very large funnel-shaped canal; terminating, in the vestibule, by an oval opening, sufficiently wide to admit the eye of a small probe. The semicircular canals were perfect; and the vestibule natural as to size and form, but communicating, by a very large opening, with a cavity somewhat resembling that on the left side, and representing the shell of a cochlea which contained the rudiment of a modiolus (that is to say, the base shutting out the cavity from the meatus internus, and allowing the passage of the auditory nervous filaments), and an attempt at the formation of a spiral lamina, which, however, did not make a complete turn, and consisted chiefly of membrane, which disappeared on the parts becoming dry from exposure. The fenestra rotunda could hardly be said to exist.

Case 2.—A boy subject to epileptic fits, died at the age of twelve years. The temporal bones were exceedingly large, massive, and heavy, and the cancellated texture was entirely deficient in the petrous portion. The meatus externus, on both sides, was partly filled with dry inspissated cerumen, and the position of the membrana tympani was nearly horizontal.

*Right Internal Ear.*—The cavity of the tympanum was completely filled with dense fleshy granulations, adhering most firmly to the walls, completely enveloping and concealing the bones, adapting themselves to all the crevices and irregularities, and extending into the mastoid cells and the commencement of the Eustachian tube. A small quantity of thin purulent fluid escaped, when the roof of the tympanum was removed. The membrana tympani was exceedingly convex, being drawn inwards until it nearly touched the promontory; thus diminishing the size of the cavity, and pushing the bones out of their place, so that the extremity of the manubrium of the malleus and the long crus of the incus were in close contact, and carried to the edge of the fenestra ovalis. No vestige of the sides of the stapes was discoverable; but it might have been broken up in the attempt to clear the tympanum from the granulations which filled it. The entrance to the cochlea presented the ordinary appearance, when viewed from the vestibule, but terminated suddenly, in a blind extremity about a line from its commencement. Of the cochlea itself no trace existed, its situation being occupied by solid hard bone. The fenestra rotunda, when traced from the tympanum, terminated in a minute cavity; which might be considered as the rudiment of a scala tympani, and into which the granulations already alluded to had entered. The auditory nerve was remarkably hard and small, and was entirely distributed to the vestibule; that portion, which in the normal condition pierces the modiolus to supply the cochlea, being, of course, deficient.

*Left Internal Ear.*—The tympanum presented nearly the same appearance as on the right side; with this addition, that the membrana was closely adherent to the promontory, producing a still greater diminution in the size of the cavity, and a greater distortion of the bones from their natural situation. The incus was anchylosed to a little spicula of bone, which projected from the inner wall of the tympanum, just above the Fallopian canal. The labyrinth presented nothing unusual, if we except, that the modiolus of the cochlea was smaller than natural, and that the granulations from the tympanum had passed through the fenestra rotunda into the commencement of the scala tympani. The auditory nerve was small and hard, by far the greater portion of it going to the vestibule.

CASE 3.—No lesion of the organ of hearing was discoverable.

CASE 4.—A girl, aged 13, died of phthisis.

*Right Ear.*—The tympanum partly filled with soft vascular granulations; a muco-purulent fluid occupying what remained of the cavity. The Eustachian tube very large, and containing a quantity of semi-inspissated mucus. The cochlea, externally, was natural, but, internally, very deficient. On tracing the scala tympani from the fenestra rotunda, it was found to take about half a turn round the base of the modiolus, and then terminated in a blind extremity. The scala vestibuli, on the other hand, formed about one-third of a turn; and then opened into a cavity which constituted the remaining part of the cochlea, as represented by its external shell. In fact, the base of the cochlea alone was perfect, the remaining portion consisting merely of the cavity just mentioned: not only was the greater portion of the spiral canals deficient, but the blind extremity of the scala tympani prevented any continuity or communication between them.

The petrous bone, more especially in the neighbourhood of the tympanum, was unusually loose and cellular, presenting numerous irregular cavities, which apparently communicated with the tympanum, and were filled with the same muco-purulent fluid: they seemed a sort of extension of the mastoid cells.

*Left Ear.*—Petrous bone irregular in shape; the meatus externus remarkably narrow, representing an elongated ellipse, when cut across. No external appearance of aquæductus vestibuli. The lining membrane of the tympanum thickened but not granular; the cavity, together with the mastoid cells and Eustachian tube, filled with soapy, adhesive, and slightly purulent mucus. The interior of the vestibule presented but four openings of the semicircular canals, instead of five; that which is common to the posterior extremity of the vertical, and the superior extremity of the oblique, being deficient. On tracing the vertical canal from its anterior opening, it was found to terminate, not by returning to the vestibule, but by becoming continuous with the upper part of the oblique canal, which latter opened below as usual. The two canals, thus united, formed one irregular tube, which extended across the superior and posterior part of the petrous bone. The cochlea presented much the same appearance as on the right side; the lamina spiralis making about half a turn round the base of the modiolus, and separating the scala tympani (which reached to about the same extent) from an irregularly-shaped cavity into which the scala vestibuli opened soon after leaving the vestibule.

CASE 5.—A boy, aged 11, died of phthisis. The entire temporal bones, but more especially the petrous portions, were most remarkable for their enormous size. In development, solidity, and weight, they exceeded any that Mr. Cock has ever seen. The external meatus was small; so were the Eustachian tubes. There was no stapedius muscle on the right side. These were the only alterations in the organ of *hearing*.

CASE 6.—A boy, aged 16, died of phthisis.

Both temporal bones were exceedingly massive, and hard in their texture; and very ill-formed, as regarded their external configuration. The tympana were irregular in shape, and their parietes more than usually rough. The Eustachian tubes were small, and irregularly contracted.

Case 7. A boy aged 12.

The examination was unsatisfactory. The bones were steeped in dilute muriatic acid for the purpose of softening them. But this altered the cochlea so completely, as to vitiate all conclusions.

Such are the cases related by our author. His physiological remarks are sensible, but contain no novelty. He sums up the appearances discovered in the preceding cases, in the following manner:—

"1. An adventitious growth filling up, and thus obliterating more or less completely, the cavity of the tympanum, enveloping the chain of bones, and encroaching upon the openings of the Eustachian tube, mastoid cells, and fenestra rotunda.

2. Deficiency of the fenestra rotunda; thus rendering one extremity of the labyrinthine canal solid and immoveable, instead of yielding.

3. Partial or complete deficiency of the spiral canals of the cochlea.

4. Preternatural enlargement of the aquæductus vestibuli.

5. Deficiency of the semicircular canals.

6. Unnatural solidity or hardness of the temporal bone."

1. His remarks on the vegetations occupying the tympanum seem to us to be very judicious.

"I think," he says, "that they would be all sufficient to occasion deafness, by preventing the transmission of vibration from the external ear to the labyrinth; while their presence at the entrance of the Eustachian tube, and at the foramen rotundum, would certainly destroy the function of two parts which are essential to the exercise of the sense of hearing. I must, however, leave the question open, as to whether this adventitious growth was coëval with birth or the period of infancy; or whether, on the other hand, it was a subsequent formation, depending upon and accruing from a prior defect in the sense of hearing, from some unknown cause;—whether, indeed, it supervened upon deafness, and was merely an effort of Nature to obliterate a cavity whose function of conveying and modifying vibration had become useless, in consequence of the previous inefficiency of that part of the organ by which the vibratory oscillations were to become converted into sound, and appreciated as such by the sensorium."

2. "With respect," he goes on to observe, "to the deficiency of the fenestra rotunda—or, in other words, the substitution of solid bone in the place of the membrane which, in the natural state, occupies that opening—such a malformation must necessarily prevent all motion in the fluid of the labyrinth, by depriving the labyrinthine canal of one of its yielding extremities, and thus cutting off from the expansion of the auditory nerve that medium of communication (viz. the motion of the water) through which it receives its impression. A similar effect will be produced where the cochlea is entirely wanting; or where the scala vestibuli and scala tympani are merely rudimentary, and do not become continuous with each other."

Mr. Cock's speculations on the effects of a preternatural enlargement of the aquæductus vestibuli may perhaps be dismissed without farther observation. Nor need we pause to weigh his remarks on the use of the semicircular canals, or the effects of their malformations. With one remark we quite coincide.

"I utterly reject the idea, that these malformations are mere coincidences, which ought not to be considered as having a reference to the imperfection or annihilation of the sense of hearing—that they are, in fact, analogous to abnormal deviations in other parts of the body, which are discovered after death, but which produced no symptoms during life, as regarded the functions of the organ in which they occurred."

While we would not have more weight attached to these cases than they will reasonably bear, we think it unphilosophical in the extreme to pronounce ex cathedrâ that such important malformations as they indicate, were unconnected with the deafness. Such dogmatic views are calculated to arrest discovery.

We hope that Mr. Cock will persevere in his laudable attempt to throw light on congenital, as well as on other forms of deafness by his pathological investigations.

### II. On the Effect produced upon the Pulse by Change of Posture. By William Augustus Guy, M. B. &c. &c.

### Influence of Sex, Age, and Period of the Day.—Experiments made with a Revolving Board.

In the last number of these Reports, Dr. Guy reported the results of experiments on the effect produced by change of posture on the pulse of healthy males. He now reports similar experiments made with the view of ascertaining how far that effect is modified by sex and age. The experiments themselves are numerous, were made with care, and are reported with minuteness. But we must content ourselves with simply transcribing their results. These are stated thus :—

" 1. In healthy females of the mean age of 27 years, in a state of rest, the number of the pulse is, standing, 89; sitting, 82; and lying, 80: the difference between standing and sitting being 7 beats; between sitting and lying, 2 beats; and between standing and lying, 9 beats. When all exceptions to the general rule are excluded, the numbers are, standing, 91; sitting, 84; and lying, 80: the difference between standing and sitting being 7 beats; between sitting and lying, 4 beats; and between standing and lying, 11 beats. The same differences, expressed fractionally, are as follow;—inclusive of exceptions, $\frac{1}{13}$, $\frac{1}{41}$, $\frac{1}{10}$;—exclusive of exceptions, $\frac{1}{13}$, $\frac{1}{21}$, $\frac{1}{8}$.

2. In females, as in males, the extreme are very remote from the mean results. Thus the greatest difference between standing and sitting is less than $\frac{1}{4}$th, the least $\frac{1}{107}$th, of the frequency standing; the greatest difference between sitting and lying is $\frac{1}{9}$, the least $\frac{1}{83}$, of the frequency sitting; whilst between standing and lying the difference may be more than $\frac{1}{4}$th, and as little as $\frac{1}{23}$, of the frequency standing.

3. The exceptions are as follow:—To the general law, that the pulse is less frequent sitting than standing, there is 1 exception in 10 experiments: to the general law, that the pulse is less frequent lying than sitting, there are 2 exceptions in 5 experiments: to the general law, that the pulse is less frequent lying than standing, there is 1 exception in 10 experiments. The total number of instances in which 1 or more exceptions to general rules occur, is 46 per cent. or nearly 1 in every 2.

4. In females, as in males, the effect produced by change of posture increases as the frequency of the pulse increases.

The following propositions relate to the differences between the male and female pulse :

1. The pulse of the adult female exceeds in frequency the pulse of the adult male of the same mean age, by from 10 to 14 beats. In the erect posture it is more frequent by about $\frac{1}{8}$th, in the sitting posture by about $\frac{1}{6}$th, and in the recumbent posture by more than $\frac{1}{5}$th.

2. Though the pulse of the adult female is more frequent than the pulse of the adult male of the same mean age, by $\frac{1}{8}$th in the erect, and $\frac{1}{5}$th in the recumbent posture, the effect of a change from the erect to the recumbent posture in the male is greater than the effect of the same change in the female by more than $\frac{1}{3}$d.

3. The effect of change of posture on any given frequency of the pulse is much greater in the male than in the female; and the disproportion is more marked in early youth than at the adult age.

4. The exceptions to the general rule are more numerous in the female than in the male, in the proportion of 4 to 3.

The following propositions refer to the influence of age:

1. The effect of change of posture is less in early youth than in the adult; and the modifying influence of age is greater in the female than in the male.

2. The exceptions to the general rule are more numerous in early youth than in the adult.

The following proposition refers to both sexes, and to all ages:—

*The exceptions to the general rule are more numerous, as the effect of change of posture is less.*"

*Influence of the Period of the Day.*

From Dr. Guy's experiments, he infers that the effect of change of posture is greatest in the forenoon and least in the afternoon; the effect in the evening being the mean between the other two; and *the effect produced by change of posture on the same frequency of the pulse in the afternoon, forenoon, and evening respectively, is as the numbers* 8, 9, *and* 10. Though the difference is not so great as it seemed to be before the varying frequency of the pulse at different times of the day was taken into account, it is sufficiently well marked to deserve attention: and if it should be confirmed by a larger number of observations, it will add to our knowledge of a very curious and interesting subject—the diurnal changes which take place in many, and probably in all, the more important functions of the human body.

*Experiments with the Horizontal Board.*

The object was to do away with that muscular exertion by which *change* of posture is effected. The subject of experiment was securely fastened to the board, and the plane of this admitted of all inclinations, between a perpendicular to the horizon *above* it, and a perpendicular to the horizon *below* it.

1. "The persons whom I submitted to experiment were conveyed from the erect posture, and supported at angles of 60°, 45°, and 30°, and then placed in the horizontal position. The pulse was carefully counted in each of these postures. Observations were made on twenty-three persons; of whom, some were Englishmen, and others German boys. I was at the time residing at Heidelberg. The mean age of the subjects of the experiment was 16 years. In the erect posture, the average frequency, in round numbers, was 89; when placed at an angle of 60°, 86; at an angle of 45°, 83; at an angle of 30°, 78; and in the horizontal posture, 75. Thus, then, the differences between each two consecutive positions were 3, 3, 5, and 3 respectively; and the total difference was 14 beats, being about $\frac{1}{7}$th of the frequency in the erect position. The difference between the results of these experiments, and of those in which the posture of the body was changed by the action of its own muscles, is, as we have already observed, less than one beat. The above correspondence between the two classes of experiments proves, indisputably, that the effect produced upon the pulse by change of posture is not due to the muscular contraction by which the posture is changed; whilst the experiments which were detailed in a former Essay leave little doubt that it must be attributed to the muscular effort by which the body is supported."

2. "The body was conveyed, as before, from the horizontal posture, through angles of 30° and 45°, to the inverted position, with the head downwards and the feet raised in the air. 21 out of the 23 persons experimented on were placed at an angle of 30°, and the pulse fell on the average 1 beat: 18 out of the 23

were placed at an angle of 45°, and their pulse fell from 77 to 76½, or ½ a beat: 12 out of the 23 were placed perpendicularly, with the head down, and the feet raised, and their pulse fell on the average 1½ beats, namely, from 78½ to 77: the total difference between the horizontal and inverted posture being 3 beats. Now this difference is so slight, that it scarcely deserves so long a notice; but when we come to examine the experiments more closely, we find that the small amount of difference is due to the great number of exceptions to the general rule of decrease. Thus, when the body was placed with the head downwards, at an angle of 30°, there were 8 exceptions in 23, or more than ⅓, in which the frequency of the pulse was increased; when placed at an angle of 45°, there were 5 such exceptions in 18, and 5 in which the pulse had the same frequency as in the former posture; and when completely inverted, there were 4 cases of increase in 12, and 2 in which the pulse had the same frequency as when the body was inclined at an angle of 45°. Now, we have already seen that, even in a transition from the erect to the horizontal position, the exceptions to the general rule were numerous; and we could scarcely expect them to be less so in the several inverted postures, even if the same causes, whatever they may be, were alone in action. But other causes came into play, amongst which, fear was not the least influential. The very novelty of the position was, to more than one of the subjects of the experiments, a source of apprehension; whilst in others, the distress produced was too great not to be accompanied by some anxiety."

"If we exclude all exceptions to the general rule, the average decrease, when the body was placed with the head downwards, at an angle of 30°, was 5; at an angle of 45°, 3; and completely inverted, 5 beats. If, in order to ensure still greater accuracy, we take those cases in which, without exception, there was a decrease up to any given position, we shall find, that at an angle of 30°, the decrease was 5; at an angle of 45°, 1; and completely inverted, 7. There were only two instances in which there was a continual decrease, without any exception to the general rule; and these occurred in two brothers who had been long in the habit of standing on their heads. In each of these the difference between the erect and inverted postures was 30 beats; the mean difference between the erect and horizontal postures being 15 beats, and that between the horizontal and inverted also 15 beats. When inclined at an angle of 30°, with the head downwards, the mean decrease was 6½ beats; at an angle of 45°, 1½; and completely inverted, 7 beats."

On the whole, then, there can be no doubt whatever that the natural tendency of the inverted position of the body is to diminish the frequency of the pulse.

Dr. Guy appears to be a very zealous and intelligent experimenter.

### III. Observations on Intus-susception as it occurs in Infants.

Mr. Gorham's attention was first particularly drawn to intus-susception, by the circumstance of a child, a few months old dying with hæmorrhage from the intestines, and intus-susception with inflammation of certain contiguous portions of the bowel being discovered after death. Not long afterwards, the following case occurred in Mr. Gorham's own practice.

*Case.* Mrs. Valence's infant, at four months and five days' age, has been healthy since birth; with the exception of having one day had ten or eleven motions, which were greener than usual. The infant has lived almost entirely on breast-milk; but, three days ago, the mother was induced to give it some panada, under the supposition that the milk was not nourishing, or in sufficient quantity. The infant was generally good, and cried but little: she appeared to be well up to yesterday morning, when at eight o'clock she passed a natural motion, and was in apparent health till two P.M., when she was sick, and vomited immediately after having taken the breast. This symptom has continued ever since. Between seven and eight of the same evening, it was first observed that the evacuations per anum consisted of blood, and nothing else: three napkins were soiled during the night, and in the morning, Mr. Gorham was requested to visit the infant.

The quantity of blood passed amounted to about three or four teaspoonsful; the skin over the entire body was pale and hot; there was no emaciation; the infant lay quiet for a few minutes; and then cried out with an expression of pain in the countenance. The pupils were dilated; there was slight cough; pulse averaged, in the half-hour, 200 in the minute; the vomited matter consisted of milk, part of which was curdled. The abdomen felt soft and hot. On introducing the little finger into the rectum, nothing abnormal could be felt; on withdrawing the finger, some dark-coloured, thickish blood followed, in quantity about a teaspoonful and a half. The infant had vomited six or eight times within the half-hour. An enema, consisting of starch and olive-oil, was now injected cold, but returned almost as fast as it was given. It was repeated with a similar result. A large poultice was now directed to be applied to the abdomen; and a quarter of a grain of extract of conium, in a teaspoonful of camphor mixture, was directed to be given every four hours, with a grain of calomel. The child passed no more blood, but she always vomited after taking the breast, convulsions came on during the night, and, at nine A.M. next morning, a fit proved fatal to her.

*Dissection six hours after death.* There were four intus-susceptions of the small intestines, which were easily reduced; and the peritoneum covering these parts was slightly red. The lower portion of the ileum was of a deep-red colour, and intus-suscepted within the ascending colon; which latter was also swallowed within the transverse arch. The appendix cœci was highly injected; and it and the cœcum were occupying the upper part of the invagination. The mucous membrane of the inflected ascending colon was beautifully injected, villous, soft, and constricted in many places; that of the invaginated ileum was intensely red, villous, and extremely irregular. The appendix cœci was of a deep reddish-brown colour, and slightly blue in many places.

The stomach was empty, and appeared to be of a healthy colour. The small intestines were generally healthy in appearance, and, for the lower three-fourths, contained a yellowish watery fluid: the upper fourth was empty. All that portion of colon which was below the intus-susception, as well as the containing parts, was of a dark-bluish colour. The mesenteric glands were enlarged, some being of the size of large beans.

Mr. Gorham, after relating this case, proceeds to offer some lengthened observations. He divides intus-susception into that unattended with inflammation, and that attended with it. Most persons are of opinion that the former, in infants, is a very common and not a dangerous affection. We shall therefore dismiss it. But this is not the case with :—

### INFLAMMATORY INTUS-SUSCEPTION.

Mr. Gorham remarks :—

"With regard to the circumstances necessary to its continuation, a remarkable difference obtains between intus-susception at the ileo-colic valve and that at any other part of the intestinal canal: for, as it has been stated that the outer fold is active, so will it continue to drag within it the *contained* intestine, provided that this latter always remain passive. And here appears to me to be the cause of that most uncontrollable invagination that occurs when the ileum is pushed into the colon : for it is not necessary that any preternatural contraction should be formed in the ileum ere it can enter the large intestine: in order to this, its normal size is sufficient. How different, on the other hand, is the case of invagination from spasm! It is in the nature of spasm to be paroxysmal—to go and come quickly: thus, affording a chance of the reduction of intus-susception, from the relaxation of spasm, and the comparative activity of the inner fold, and this in a direction contrary to that of the invagination. Intus-susception, then, at the ileo-colic valve, occurs without spasm, without a preternatural contraction of intestine, and, consequently, without a chance of reparation from any subsequent dilatation that might have occurred, had spasm existed."

Mr. Gorham presents a table, which gives in a concise form, the particulars of nine cases of intus-susception occurring in very young infants. This table he analyses, and offers the principal inferences that may be drawn from the facts on which it is founded.

*Symptoms.*—In all the cases on record, with two exceptions, the child is said to have been in good health prior to the attack.

Vomiting was almost a constant symptom. The pulse did not appear to afford sufficient grounds for any satisfactory inference. A tumor could be detected in the abdomen in some of the cases. There were strong indications of pain, such as violent paroxysmal screamings, drawing up of the legs, and elevation of the upper lip, &c.—The constant symptom, however, was, the passing of blood per anum, in various degrees of purity; never indeed contaminated with feculent matter, but chiefly with mucus. The blood appears to be recent, and fluid, in many cases; and in quantity varies considerably.

"In Mr. Muriel's case it was excessive, more than a teacupful of blood having been passed. The disease, in fact, amounted to hæmorrhage from the intestines, of which the causes were not known before death; insomuch that the infant was completely blanched and cold ere the fatal termination occurred. In my own case, I must say that I was struck with the appearance of this sort of discharge per anum, in a patient so young. It was not that of common dysentery; where the intestine seems reluctantly to supply its blood, to tinge the mucus which is generally present in that disease. It came on suddenly in infants who had previously enjoyed good health. It had nothing to do with diarrhœa; for this was not, neither had been, present in any of the cases. Neither had it occurred during a peritonitis; in which disease, sometimes, blood is passed. Purpura was not present; for this is rare in infants so young; neither could hæmorrhoids or organic disease be detected."

"In all the fatal cases reported, the same intestine, viz. colon, had formed the *containing* fold; and this had grasped the *contained* parts, (viz. the lower portion of the ileum, the vermiform process, the cœcum, and more or less of the inverted colon), which it urged onwards by its natural peristaltic motion, as if it were endeavouring to expel them by the anus. This it had almost accomplished, in Dr. Lettsom's case: in my own, the contained portions had not progressed so far: in Dr. Ash's case, Mr. Blizard's, and Mr. Langstaff's, it had proceeded as low as the sigmoid flexure. Nor has the effort only been made, but the expulsion has actually been accomplished in a most astonishing manner; for the invaginated portion has sometimes sloughed, and been discharged per anum, while the agglutination of the parts has preserved the continuity of the intestinal canal. Thus, in a case related in Duncan's Commentaries, eighteen inches of small intestine were voided per anum. Three similar instances occur in M. Hevin's Memoir; 23 inches of colon came away in one of them, and 28 of small intestines in another. Other cases occur in the Physical and Literary Essays; in Duncan's Annals; and in the Medico-Chirurgical Transactions, where Dr. Baillie states that a yard of intestine was voided. In 1823, M. Bush recorded a case, in which from 15 to 18 inches of the ileum were discharged from the anus; and recovery was effected on this principle."

Though all ages may be affected with the disease, yet infancy and childhood are particularly subject to it. In adult life the symptoms are more variable. Thus, in a case reported by Mr. Bullin, of Fleet Market, of an adult, in which the ileum and cœcum were found invaginated within the colon in a manner precisely similar to that of those mentioned in this paper, the chief symptoms were, suppression of stools, and violent pains in the abdomen, quite unattended with vomiting.

As has been already stated, the most frequent seat of the affection is at the termination of the small intestine in the cœcum. The next most usual situation is the ileum—the least the jejunum.

The immediate cause would seem to be spasm of the invaginated part of the intestine. The exciting causes are probably such as would produce that spasm.

The characteristic symptoms have been pointed out, but may be recapitulated:

When an infant under a year old is seized with symptoms of strangulated hernia, the cause will most frequently be *intus-susception.*

When dangerous intus-susception exists, its situation will most frequently be at the termination of the ileum in the cœcum.

Hæmorrhage, with absence of all fæcal evacuations from the intestine of an infant, is rare, unless it have for its cause *intus-susception.*

We will not say that Mr. Gorham has totally avoided the fault of generalizing very boldly on premises which cannot be considered sufficient to establish positive conclusions.

*Treatment.*—Bleeding, quicksilver, forcible clysters, a long bougie, anodynes, strong purgatives, the warm bath, blisters, emetics, have all been recommended and practised, some with more and some with less advantage. It has been proposed to open the abdomen, and disentangle the intus-suscepted gut—a proposal which may have been acted on once or twice with success, but which, if generally adopted would certainly be most hazardous. Mr. Gorham speaks with favour of the method of treating the disease by *inflation.* This is effected by introducing the nozzle of a common bellows into the rectum, and gradually blowing up the intestines. Three cases in which this plan succeeded have been reported in the American Journal of Medical Science. The following is a condensed account of them.

*Case* 1.—The patient was a female, aged 26. After having uneasy sensations in the stomach, obstinate vomiting succeeded, which consisted, at a subsequent period, of yellow matter. A violent screwing pain was also complained of, situate between the sternum and umbilicus: it came on in paroxysms, and ended in vomiting. Calomel, jalap, castor-oil, laudanum, the warm-bath, and effervescents, were all useless; and after five days passed without a dejection, the common bellows was used; and it is stated, that "as soon as air entered the rectum the countenance lost its anxiety, and the patient said she felt quite relieved. In a minute she passed a stool; and complete recovery resulted."*

*Case* 2.—The patient was a male, aged 35. The symptoms were, first, dry retching, and hiccup; afterwards, vomiting of a large quantity of green bile, mixed with feculent matter: violent pain was complained of in the umbilical region; the pulse was small, frequent, and irregular. Superficial examination detected no peculiarity in the form of the abdomen, till the fourth day, when an unusual fulness and firmness was first discovered in the right iliac region; but the hand lying upon the spot, a paroxysm of pain occurred, and an elongated tumor was felt to rise, with an erectile motion. Purgatives of croton-oil combined with laudanum, fomentation, enemata of tobacco infusion, and copious

* American Journal of Medical Science, XXVI. 542.

bleeding, were all of no avail; and on the fifth day, as a last resource, the bellows was used. After the first inflation, there was no occurrence of violent pain; the patient said he felt much easier, and wished to pass a motion: a large quantity of air, however, came away, and about a gill of very fetid bloody water. In about five hours after this, two copious dejections were passed; and complete recovery ensued.*

*Case* 3.—The symptoms were, vomiting of a dark, fetid, oily fluid; hiccup; with severe pain round the navel: no motion was passed for four days, at the end of which time the bellows was used. Six dejections followed in the course of the day. The patient recovered.

The method should be tried in any severe case. It cannot well do harm, and although it is by no means certain that the preceding cases were examples of intus-susception, yet they are calculated to give encouragement.

"Mr. Finch, a general practitioner, residing at Greenwich, informs me he has treated cases successfully in the following manner. Injections of warm thin gruel are used; and if any advantage be expected to be derived from them, they must be prevented returning. In order to this, Mr. Finch causes the pipe to assume a conical shape, by binding lint, or some soft material, round it. The piston is then pressed with considerable force; and the return of the intestine is known to have taken place by the want of resistance suddenly communicated to the hand. Mr. Finch has treated two cases successfully in this manner."

The action of the enema would be similar to that of inflation. But, probably, the latter would be most effectual.

Mr. Gorham's paper is not undeserving of perusal.

Dr. G. O. Rees communicates to this number of the Reports, two chemical analyses—one of the liquor amnii—and one of diabetic blood.

### IV. Chemical Examination of the Liquor Amnii. By G. O. Rees, M.D. &c.

The analyses hitherto made of this fluid have varied. Dr. Rees has examined it in four instances. It was procured with all precautions for its purity by Mr. C. W. Lever.

1. Labour induced at seven months and a half, by passing a female catheter through the os uteri, and drawing off the liquor amnii.

EXAMINATION OF LIQUOR AMNII.

Strongly alkaline.—Sp. grav. 1008.6.

Contained in 1000 parts:

| | |
|---|---|
| Water .. .. .. .. .. .. .. .. .. | 983.4 |
| Albumen (traces of fatty matter) .. .. | 5.9 |
| Albuminate of soda } Chloride of sodium } .. .. .. .. .. | 6.1 |
| Animal extractive soluble in water and alcohol, urea, chloride of sodium .. .. | 4.6 |
| Traces of alkaline sulphate. | |

2. Patient died of phthisis at about the seventh or eighth month of gestation.

EXAMINATION OF LIQUOR AMNII.

Strongly alkaline.—Sp. grav. 1008.

Contained in 1000 parts:

* American Journal of Medical Science, XXX. 556.

| | | |
|---|---|---|
| Water .. .. .. .. .. .. .. .. .. .. | | 984.98 |
| Albumen (traces of fatty matter) .. .. .. .. .. | | 1.80 |
| Extract soluble in water | Salts 2.80<br>Organic matter, principally albumen, from albuminate of soda, 3.22 .. .. .. | 6.02 |
| Extract soluble in water & alcohol | Salts 2.80<br>Organic matter, principally lactic acid and urea, 4.4 .. | 7.20 |

3. Again obtained at seven months and a half, from patient No. 1, by the same means.

EXAMINATION OF THE LIQUOR AMNII.

Strongly alkaline.—Sp. grav. 1007.

Contained in 1000 parts:

| | | |
|---|---|---|
| Water .. .. .. .. .. .. .. .. .. .. | | 986.8 |
| Albumen (traces of fatty matter) .. .. .. .. .. | | 3.2 |
| Aqueous extract | Salts 3.2<br>Organic matter, viz. albumen, from albuminate of soda, 1.2 | 4.4 |
| Alcoholic extract | Salts 3.8<br>Organic matter, viz. lactic acid and urea, 1.8 | 5.6 |

4. Labour induced at seven months and a half, and liquor amnii drawn off by a female catheter.

EXAMINATION OF THE LIQUOR AMNII.

Strongly alkaline.—Sp. grav. 1007.

Contained in 1000 parts:

| | | |
|---|---|---|
| Water.. .. .. .. .. .. .. .. .. .. .. | | 986.8 |
| Albumen (traces of fatty matter) .. .. .. .. .. | | 2.4 |
| Aqueous extract | Salts 4.2<br>Organic matter, viz. albumen, from albuminate of soda, 2.0 | 6.2 |
| Alcoholic extract | Salts 3.0<br>Organic matter, viz. lactic acid, and urea, 1.6 .. .. .. | 4.6 |

"The salts, both of the aqueous and alcoholic extracts, consisted of chloride of sodium and carbonate of soda, with minute traces of an alkaline sulphate and phosphate. In the salts of the aqueous extractive, the carbonate resulted from the incineration of an albuminate; and in the alcoholic extractive, from the decomposition of a lactate, by the same operation.

The salts obtained from the aqueous extractive in analyses Nos. 2 and 3 were not entirely soluble in water. The insoluble matter, on examination, proved to be phosphate of lime; which must either have been held in solution with the albuminate of soda, or have resulted from the decomposition of an alkaline phosphate at a red heat; some soluble earthy salt being present, to effect such decomposition. I lately observed the existence of phosphate of lime in the aqueous extractive of a specimen of blood drawn from a diabetic patient; and am inclined to think that further observations will shew a similar result in other albuminous fluids."

The salts observed floating in the liquors are composed of caseous matter, containing cholesterine.

"On examining the analyses, it will be observed that the liquor amnii varies greatly in proportional constitution in different individuals, at the same period of utero-gestation; which shews, that, like perhaps all the secretions of the body, it is affected by the temperament and diathesis of the mother. The specific gravity of the secretion, however, varies but little in the four specimens; which is possibly a precaution on the part of nature to preserve a medium of fixed power, to oppose the motions of the fœtus in utero."

### V. ON DIABETIC BLOOD. By G. O. REES, M.D. &c. &c.

It is only lately that the presence of sugar has been demonstrated in diabetic blood, a fact that was long denied by chemists. Mr. M'Grigor, of Glasgow, has shewn, or seemed to shew, that sugar is present, not only in the blood and urine, but likewise in several secretions and excretions. Ambrosiani relates a method by which he succeeded in extracting it in a crystalized state. But the mode which Dr. Rees has adopted will yield sugar of considerable purity, though it will not enable us to determine with precision the weight. We shall therefore present an account of this plan of procedure :—

" The mass of blood* is to be evaporated to dryness, over a water-bath; the dried mass to be comminuted, and digested for several hours in boiling water: the aqueous solution is to be filtered off, evaporated to dryness, and the dried residuum digested in alcohol of sp. gr. 0.825: the alcoholic solution so formed is to be filtered, or carefully poured off, evaporated to dryness, and the dry mass treated several times with rectified ether, which dissolves out urea, and also some fatty matter; leaving behind the sugar, in admixture with osmazome and chloride of sodium: this mass, on being dissolved in alcohol, and the solution allowed to evaporate spontaneously in a flat glass dish, affords mixed crystals of alkaline chloride and diabetic sugar; which are easily distinguishable from each other, and allow of being separated mechanically, by shaking them up in alcohol, when the chloride sinks; and the sugar, being principally collected above, may be removed, for examination, by careful use of the spatula: the alcohol must not, of course, be allowed to remain long in contact with the crystals, as it would re-dissolve them. It is a matter of surprise to me, that sugar has not been long ago detected in the blood of diabetic patients, though not separated from it; for the alcoholic extract of the serum, when mixed with water, will, after a few days, give off carbonic acid; which, in addition to the sweetish taste, and, I may add, syrupy smell of the evaporated alcoholic extract, is a sufficient evidence of the presence of sugar. I subjoin the analysis of 1000 grains of diabetic serum, obtained for me by the kindness of Dr. Bright. The sp. gr. of this patient's urine was 1048; and the contents of the serum as follows:—

| | |
|---|---|
| Water | 908.50 |
| Albumen (yielding traces of phosphate of lime and oxide of iron, on incineration) | 80.35 |
| Fatty matters | 0.95 |
| Diabetic sugar | 1.80 |
| Animal extractive, soluble in alcohol, urea | 2.20 |
| Albuminate of soda | 0.80 |
| Alkaline chloride, with traces of phosphate<br>Alkaline carbonate, and trace of sulphate, the results of incineration | 4.40 |
| Loss | 1.00 |
| | 1000.00." |

* "12 ounces were used in these experiments."

Dr. Rees observes that, on comparing this analysis with that of the serum of healthy blood, we perceive a great excess of matters soluble in alcohol, while the albuminate of soda is rather less than in health. The alkaline salts are also in very small proportion, being only 4.40 gr, in 1000 grains of serum, while in health they amount to from 7 to 8 grains per 1000.

### VI. Some Observations on the Cause of Strangulation in Hernia, and on the Causes of Death, and also on the Rules of Treatment, which such Considerations enforce. By T. Wilkinson King.

This Paper, which displays much industry and reflection on the part of Mr. King, contains an application of the numerical method to the elucidation of some points connected with Hernia.

There is a table of 100 cases, compiled from the best authorities, which required surgical aid on account of urgent syptoms. The principal deductions from the data thus collected and compared are as follows :—

1. *Most herniæ exist for years, before they become subject to dangerous strangulation.*

Thus on analysing the Table, we discover that:

In 2 cases, the duration is not mentioned.
.. 3 or 4 cases we might determine, at once, that acute strangulation was the marked condition.
..15 cases, the hernia is said to be "old."
..18 cases . . . . . . . . of "some, several, or many years'," duration, or to be "adherent in the sac."
..46 cases, the duration is defined in years; and the mean for the whole of these is about 18 years. The minimum, about 4; and the maximum, 44 years.
..15 cases, the duration may be inferred within a year or two: and the mean for these I make to be 25 years. The maximum near 60.

100

Hence, of 61 cases, the mean duration is about 20 years; and of 33 cases more, we may say they were indefinitely old: so that 94, out of 98, were in various degrees, "old."

Making every allowance for some imperceptible source of fallacy, it will be readily admitted that the antiquity of herniæ which become strangulated is surprizing. Mr. King advances some ingenious reasons for concluding that the cause is to be found in the morbid states and diminished vital powers of the old herniary protrusion. There is, no doubt, much force in this, whether it does or does not explain the whole of the phenomena.

2. *Causes of Death.* These are calculated from unpublished records of above forty fatal cases of hernia. In the majority of instances the patient died of peritonitis.

The following is a tabular view of thirty-eight cases.

In 1, the hernia was reduced by taxis: death by *peritonitis:* the gut red.
1 .. .. .. .. .. .. *peritonitis ?* recovering.
1 .. .. .. .. .. sac and all, the stricture remaining.
1 .. .. returned by operation .. .. .. ..
3 .. .. was not returned: no operation: no gangrene: *peritonitis.*
6 .. .. was returned by taxis, but ruptured: death by *peritonitis.*
12 .. .. .. .. operation: *peritonitis;* the portions variously dark.

4 .. .. .. .. .. .. .. .. gangrene extensive.
8 .. .. was exposed: not returned: artificial anus, or the like: death by *peritonitis.*
1 .. .. was returned: the omentum divided: death by *internal hæmorrhage.*

The object of Mr. King is to dwell, which he does, on peritonitis, in a low and unhealthy form, being the prevalent cause of death. On this he insists. He remarks, too, that in the fatal cases, there are usually organic changes of more or less importance in the great viscera—such changes as occur after the meridian of life, and such as, though not incompatible with seeming health, are, by no means favourable to any material reparative effort.

Yet we hardly know what to say to this strong position of Mr. King's. The general opinion with respect to the issue of most fatal cases of hernia after operation, is, that death is owing to a sort of " paralysis " of the bowel. A part of it is found dark, and the general evidences of peritonitis, to any extent, are absent. We must own that this hypothesis has never been very satisfactory to ourselves, and we are rather inclined to attribute death to low inflammatory action, and the depressing influence on the system of a portion of gut seriously injured.

Mr. King makes some observations on the treatment of hernia, at which we may just glance.

" In the *reduction of herniæ,* I have certainly seen degrees of force successful that I should be very unwilling to use: and if it be near the truth, that about one-fourth of the deaths by hernia follow the simple taxis, it may be allowed that there is less reason to trust in this means so implicitly as we are taught, whether we regard the employment of force or not. Of course, nothing can ever militate against the early and careful use of the taxis: but may we not conclude, that delay, repetition, and violence, in connexion with the actual return of hernia by taxis, would appear more reprehensible than is commonly thought?

I find included in the Table of Fatal Cases, eight in which the taxis was the cause of death. One was a reduction *' en bloc :'* a ninth was the same thing (but for the operation): and one only, I think, of the six in which the bowel was ruptured, was connected with some previous accidental injury. One case was a merely dark bowel, well returned: a tenth case was similar, but erysipelas complicated the peritonitis: and, if my memory does not betray me, I have also known one or two other cases of fatal taxis, in which the bowel was returned entire, and no distinct gangrene was discoverable after death."

We may observe, that a great improvement has taken place, of late years, in the treatment of hernia in reference to this very point. The taxis is neither so much nor so long employed as formerly. Still we think, with Mr. King, that even at present it is used too often, too long, and too forcibly. The great reason for its being so is, probably, the general reluctance on the part both of surgeon and patient to the knife. Yet there can be no question that the success of the operation has been much in the ratio of its early performance.

Mr. King enters a strong protest against the use of purgatives after the operation. This is a difficult point to decide, for a good deal is to be said on both sides. We do not mean that any thing rational can be said in support of incessant or violent purgation, but we are not quite so sure that, when the bowel does not soon evince a tendency to act, gentle aperients are objectionable in theory or injurious in fact.

Mr. King concludes, and so shall we, with the following summary:—

" First—Most herniæ being of old standing before they become seriously strangulated, this result is not attributable to the state of the sac, but to that of the bowel; in which, defective nourishment and power of vessels leads to more ready tumefaction;—and all this seems attributable to the age and the organic deterioration belonging to it.

2dly—The common and chief danger is from a peculiar and unhealthy kind of peritonitis; the consequence, probably, of the same constitutional decay or decline of organs which induced the strangulation.

3dly—The above facts lead to the conclusion, that prompt surgery, to remove the cause of inflammation, and the most cautious medicine to obviate and not excite inflammation, and to add nothing to the oppressed condition of the patient, are indications even more pressing than has been commonly maintained, at least among authors and the generality of surgeons."

### VII. Cases and Observations in Medical Jurisprudence. By Alfred S. Taylor.

These are on Poisoning by Oxalic Acid—on Rupture of the Diaphragm—and on Open Foramen Ovale.

We see nothing in the Report of a case of poisoning by oxalic acid to call for particular notice.

#### Survival after Extensive Rupture of the Diaphragm.

Rupture of the Diaphragm has been pronounced mortal, and a very exact and formidable train of symptoms have been chronicled as appertaining to it. With what truth unqualified observations of this kind have been made or are to be taken, may be judged from the following case.

E. L. aged 40, a sailor, was admitted into the hospital, March 7, 1838, under the care of Mr. Morgan. His look was healthy, his body spare; but his general health had been always pretty good. Six months previous to his admission, he had fallen on the deck of a vessel, from a great height; in consequence of which, his ankle was severely injured, and his ribs were fractured. He suffered most from his ankle: a portion of bone had come away, and it had continued bad up to the present time. When examined, it was found to be in a sloughy state; and for this he was admitted. He did not complain of much pain; his appetite was good; and his bowels were regular.

His ankle became so much worse that amputation was performed on the 2nd of May, and in spite of all attentions, he sank, and died on the 4th of June.

Subsequently to the operation, his chest was examined several times with the stethoscope, and the respiratory murmur was found rather deficient at the apices of both lungs; but still there was nothing remarkable in the sound of his voice, nor in the act of respiration before death. The chest, on percussion, gave out a good sound everywhere, except over the lower part of the left lung. The deceased stated, that many years ago he had met with an accident, by which he had fractured some of his ribs on the left side.

*Dissection.*—" When the chest was opened, the heart was observed to be small, and to be situated rather to the right of the median line. The most remarkable and unexpected appearances were seen on the left side of the chest. The left lung, which was much contracted, lay superiorly, and behind. Its upper portion was pale, doughy, and crepitant: its middle portion, unusually divided from the rest, was pale, soft, and completely hepatized. Inferiorly, this organ was of a dark red colour, empty, and compressed. The cause of this abnormal condition now became obvious. Two thirds of this side, or nearly one half of the whole cavity of the chest, was filled up by the distended stomach, and a long curve of the arch of the colon. These parts, which had protruded through an aperture in the diaphragm, were covered with a great deal of thin omentum. It was found, on further examination, that the opening, which was two-and-a-half inches in extent, was situated in the muscular part of the diaphragm, anteriorly, and a little to the left of the œsophageal opening. The margin of the aperture, to which the omentum was in one or two places strongly adherent, was opake, yellowish, firm, and even. The cardia was just within the opening, and the pylorus also posteriorly. The ascending colon ran in on the right side, and a little posteriorly; while the descending colon ran out anteriorly. The bowel, in a contracted state, to the extent of about twelve inches, was folded vertically, on the right end of the stomach, which was much distended with air. This organ contained some dark-coloured liquid: its surface was reddened, and a good deal softened. It was covered externally, in great part, by the omentum. The fifth rib had been fractured near its middle, and the broken end had pierced the two layers of pleura:—patches of fibrin were found on the lung. The omentum adhered to the broken rib and surrounding parts. The third rib had been fractured, and much displaced. The fourth and sixth ribs were simply broken, and united. These injuries were of old standing. The viscera of the abdomen presented no particular appearances."

### Open Foramen Ovale.

It is now pretty generally understood that patency of the foramen ovale may exist without any obvious symptoms. If there be no other malformation it is probable that little or no inconvenience would ensue, unless cardiac disease and interruption of the balance between the respective portions of the organ supervene. Mr. Taylor relates two cases, in one of which there were no symptoms, and in the other there were indistinct ones; but in the latter, the pulmonary artery was small, and the left ventricle more developed than the right. We examined the body of an adult the other day, who had presented, so far as we could learn, no symptoms of cardiac disturbance, and in whom the foramen ovale was quite open; the finger could be passed from one auricle to the other with facility.

Mr. Taylor quotes some observations of Billard's, with which we shall conclude:

"Billard's observations, made on 89 cases, have entirely deprived this change of all value, as a sign for the determination of the survivorship of a child. Thus, he found the opening entirely closed—once in 18 subjects of one day old—four times in 22 subjects of two days—three times in 22 subjects of three days—twice in 27 subjects of four days. Hence we see that it was found more frequently closed on the second and third days—*i. e.* in $\frac{1}{5}$th and $\frac{1}{7}$th of the cases—than on the first and fourth days—*i. e.* in $\frac{1}{18}$th and $\frac{1}{13}$th of the cases. To make this change of any use, as evidence of survivorship, the cases of its closure ought to increase, in a uniform ratio, in proportion to the period which the child lives after it is born: but here we see, that it was closed twice as frequently on the *second* and *third* days, as on the *fourth*."

### VIII. Observations on Abdominal Tumors and Intumescence: Illustrated by Cases of Disease of the Spleen. With Remarks on the General Pathology of that Viscus. By R. Bright, M.D., F.R.S., Physician Extraordinary to the Queen.

We are glad to perceive that our able friend Dr. Bright still contributes zealously to these Reports. His present paper, as may be seen from its heading, is dedicated to the pathology of the spleen.

Dr. Bright observes very pertinently that both the situation and the obscurity attending the functions of the spleen conspire to render the diagnosis of its morbid alterations difficult. He gives a sketch of the position, structure, and probable office of the viscus, on which we see no necessity for entering.

The alterations of the organ to which Dr. Bright adverts are:—

### A. Alterations of the Substance of the Spleen.

1. *Simple Congestion.*—The result of over-distention, and of accumulation of blood in the texture of the spleen to that extent which the proper tunic and the peritoneum will permit.

The spleen, in this case, retains its natural structure; and is, for a time at least, capable of being completely relieved. This condition of the spleen is probably often produced by repressed perspiration, and sudden or long-continued cold: it occurs, in a more permanent way, after some continuance of intermittent fever.

2. *Congestion with Enlargement, and probably partial Organic Change—the Spleen still apparently Able to Discharge its Function.*—In this state of disease, although the viscus is obviously and often greatly enlarged, yet, from the fact, that the constitution does not materially suffer, that the countenance remains healthy, and that the spleen is subject to occasional fluctuations as to size, there is reason to believe that portions, at least, of the viscus, and most likely the whole, partially admits the usual passage of the blood.

3. *Fleshy Hardness.*—The organ is completely altered in its texture and characters: it becomes firm to the touch, cutting with as much resistance as an half-ripe apple, and the cut surface yielding the lustre of a firm damson-cheese; and sometimes, the cut surface presents numerous opake whitish granules, apparently from thickened cellular membranes. It is probable that this condition is the result of chronic inflammation, or of frequent congestion; but as the spleen is often not materially enlarged, it happens that, in the present state of our knowledge, we have no means of ascertaining its induration during life.

4. *Fleshy Hardness, with Enlargement.*—In this state, the spleen often attains to a prodigious size, filling up the whole left side of the abdomen. It produces very little constitutional irritation, and chiefly injures by its bulk, and its tendency to favour serous effusion. It is astonishing with what rapidity this enormous growth occasionally takes place; but in this respect we are liable to be deceived, for it is attended by so little pain, that, in many cases, the increase has been taking place, gradually, long before some accidental circumstance leads to its discovery. In young children this form of disease is more frequent and fatal than in adults. It often begins at the age of two or three months, and may attain a great size, being traceable into the pelvis, and far to the right side. It is often attended with the appearance of petechiæ all over their cadaverous and pale bodies. Such children seldom live above a year, or two or three; and fall victims to emaciation, and often to mesenteric disease.

5. *Softening.*—"This condition may exist in various degrees, and may depend on different causes. It is sometimes rather the result of rapid change after death than of disease. Where the colour of the organ is that of a deep venous blood, probably congestion and cadaveric change may be inferred; and where a lighter lilac colour, or a mottled red and lilac, is observed, it has been supposed to bespeak some form of inflammatory action."

6. *Inflammation.*—"How much of the enlargement, and the permanent hardening and softening, of which I have just spoken, may be the result of inflammation, and how much of congestion often repeated, may be matter of doubt; but that the spleen is subject to inflammation in its substance, like other organs, is certain: and although, from its peculiar character and colour, it is not easy to point out its appearance under recent inflammatory attacks, we have reason to suppose that certain alterations, with regard to its general vascularity and its consistence, must be so produced; and, perhaps, as I have said, the red and mottled lilac colour, accompanied with a degree of turgescence in the organ, indicates a state of inflammation."

7. *Suppuration.*—Dr. Bright has seen this two or three times. The spleen, under such circumstances, is apt to contract adhesions with neighbouring viscera; and either form a kind of shut sac by their assistance; or ulceration may effect the discharge of the abscess into some of the hollow viscera.

8. *Gangrene* occasionally happens.

9. *Tubercles.*—"The substance of the spleen is occasionally sprinkled with genuine tubercles. They are often very regularly distributed through the whole substance; and, whether more or less frequent, seem to occupy every part equally. They are sometimes solid and hard; but very soon incline to soften at their centres, and early present the appearance of curdled matter contained in little cysts. The tubercles in the spleen are generally, but not always, accompanied by similar deposits in other organs, particularly the lungs and mesenteric glands: and the tubercular diathesis, in very young children, more frequently shews itself in the spleen than in persons of more advanced age; and seems to bear proportion rather to the disease of the glandular system, than of the lungs."

10. *Malignant Disease.*—Both the scirrhous and cerebriform tuber sometimes appear in the spleen. It displays its usual tendency to form rounded masses, and gradually insinuates itself extensively into the substance of the organ.

11. *Melanosis.*—A fine preparation of this deposit in the spleen is exhibited in the military museum of Fort Pitt. We have seen a case of the same kind.

12. "There is another form of disease, which appears to be of a malignant character, though it varies from the more usual forms of malignant disease; and which has been particularly pointed out by Dr. Hodgkin, as connected with extensive disease of the absorbent glands, more particularly those which accompany the blood-vessels. The whole of these absorbent glands, or large masses of them, become large and firm; without any tendency to suppuration, as in ordinary scrofulous disease; or to soften, as in cerebriform disease; and, at the same time, the spleen becomes more or less completely infiltrated, throughout its whole substance, with a white matter of almost the appearance of suet. This matter insinuates itself into the cellular structure of the spleen; but it is no easy matter to point out what particular portion of the structure receives it. A section of the organ seems to shew, from the irregular forms assumed, that it fills a cellular structure, and, in some degree, takes its shape from the cells into which it enters; having less tendency to assume the form of regular globular masses or tubera than other malignant disease."

13. *Fibrinous Deposits, most probably from Extravasated Blood.*—In several cases, says Dr. Bright, it has been observed, in the examination of bodies, that the spleen has presented, when first brought into view, in the middle of its structure, a large mass, or sometimes two masses, of a yellow fibrinous matter, of uniform consistence. In some cases, these have had all the appearance of being the remnants of blood, thrown out, either like an apoplectic clot, or from the rupture or laceration of the spleen: they have sometimes presented, towards their edges, some appearance of an unconverted clot: they have not appeared to be in any state of active progress: they have generally been larger on the part seen externally, diminishing inwards, as might be expected if they had filled up a fissure or rent in the substance. In some cases, the tunics of the spleen have not been distinctly traceable; in others, they have seemed uninjured.

14. *Bony Deposits.*—Small rounded masses of bone are now and then found in the very centre of the spleen. In the only case that Dr. B. has seen, he found them also in the mesenteric glands. This coincidence is the more remarkable from similar coincidences in the case of malignant disease, and of the peculiar deposit just described.

15. *Cellular Degeneration.*—"There is an occasional appearance presented by the spleen; which I have also seen both in the liver and the kidney, where cells are developed, as if in the cellular membrane, filled with serous fluid. From these appearances, we should be inclined to suppose that they were of little importance, and only likely to interfere with the functions of the organ when they have occupied a much larger portion of it than I have ever witnessed."

16. *Hydatids.*—Not so often witnessed in the spleen as in the liver.

17. *Laceration.*—An occasional result of violence, and more likely to happen if the spleen is turgid at the time of the injury.

"There are instances of laceration taking place without external injury; and the late Dr. Babington related to me a case where he had examined a patient after death, in whom the spleen had been completely detached, and was found loose in the pelvis. In that case, most violent sickness had taken place; and was believed to be the cause, not the consequence, of the spleen being torn from its attachment."

18. *Supernumerary Spleens.*—These are common enough. Dr. Bright has never seen a deficiency of spleen altogether.

19. *Purulent Deposits in the Spleen.*—We observe that Dr. Bright makes no mention of these. They cannot be viewed merely in the light of ordinary suppuration. We have seen such deposits several times.

### B. Ulcerations of the Tunics of the Spleen.

The peritoneal covering of the spleen, like the peritoneum elsewhere, may be vascular, from inflammation or congestion: it is subject to ecchymosis, adhesion, fibrinous deposit on its surface, cartilaginous deposit, and bony deposit. It may also be covered with tubercular matter, and with different forms of malignant growth.

1. *Vascularity.*—This is not so obvious, nor so common, as in many other parts.

2. *Ecchymosis.*—This is most frequently the result of accident, blows, and falls, accompanied by contusions or lacerations of the organ: but, besides this, it is sometimes seen as the result of disease; as when, in some cases of dropsy, the peritoneum has extensively put on the hæmorrhagic tendency; and this is more particularly favoured by organic changes in the spleen and liver.

3. *Adhesion.*—A common consequence of inflammatory action in the spleen.

4. *Fibrinous Deposit.*—The surface of the spleen is often found covered with fibrinous deposit, when inflammation has existed in the peritoneum; for, owing to its dependent position when the patient is lying on his back, it happens that the deposit has an opportunity of accumulating about it, and fixes itself permanently upon it.

5. *Cartilaginous Deposit.*—"The frequency of this occurrence is somewhat peculiar to the spleen; for there is no other organ so often found to be covered with a partial cartilaginous coating. The convex surface is the most frequent seat of this appearance. Sometimes the whole is covered with an even, shining coat of cartilage; at other times, it is distributed in masses of larger or smaller size, with intervening spaces; and sometimes it will be found nearly a quarter of an inch in thickuess. This deposit seems to belong to the peritoneal coat, in the substance of which, or on its substance, it is probably formed: and it does not materially interfere with the elasticity of the proper tunic; so that I have seen a spleen covered with a coating of cartilage of extraordinary thickness, which had contracted so as to bring the cartilaginous covering, which was incapable of contracting, into numerous folds. It is probably owing to the situation of the spleen favouring the accumulation of fibrin on its surface, and the peculiar and constant motion to which it is subject, in accordance with the motion of the stomach, and its own distention and contraction, that these cartilaginous patches are so frequent on its surface." 408.

6. *Bony Deposit.*—Occasionally an extension of the preceding change leads to the formation of plates and spicula of bone in portions of the cartilage, or even of a complete bony case.

7. *Cicatrices.*—It is not unusual to see appearances like scars, from healed lesions, upon the surface; and these sometimes penetrate quite into the substance. There is no reason to doubt that these are, then, results, either of accidental injuries, or of inflammatory and, in some cases, perhaps, suppurative action.

8. *Tubercular Deposit.*—When the tubercular diathesis is strong, and the serous membranes take on the action, or, as more frequently happens, when the newly-deposited products of inflammation receive the tubercular deposit, the spleen is often covered, either by a coating of such matter, or by innumerable miliary tubercles.

9. *Malignant Deposit.*—The peritoneal coat of the spleen, and the cellular membrane beneath it, receive the different forms of malignant growth; sometimes the pendulous cysts and tubera, sometimes the creeping flat circular deposits, and sometimes the general even deposit of this kind.

Such is the circumstantial account which Dr. Bright presents of the morbid alterations of the spleen and its envelopes. He proceeds to offer a sketch, of necessity a brief one, of their symptoms.

### C. SYMPTOMS.

Dr. Bright observes, that, as we are ignorant of the function of the spleen, functional disturbance, the usual clue to organic alteration, lends us no assistance here. But, he goes on to remark:—

"From experience we know, that, not unfrequently, certain splenic diseases are concomitant with a peculiarly unhealthy, sallow, and anæmial character of countenance: we also know, that, not unfrequently, an hæmorrhagic tendency is an accompaniment of such disorders: but neither of these are sufficiently defined; nor are they sufficiently limited to affections of the spleen, to furnish more than a clue in the investigation: and probably the local symptoms are those to which we shall turn with the greatest advantage. These local symptoms are pain, tenderness on pressure, and tumors. The pain is seldom acute, unless the peritoneal coat of the organ is inflamed; and then, owing to the proximity of so many other parts, as the heart, the lungs, the diaphragm, the stomach, the kidneys, the colon, it is very difficult to localize; although, by the method of abstracting one by one of the organs, in proof of the lesions of which certain other symptoms are wanting, we may come to the conclusion that the pain belongs to the spleen. There is a dull and tensive pain sometimes complained of; but this, in general, does not occur till the tumor is already capable of being felt. The tenderness induced by pressure seldom leads us to the exact seat of disease, before the situation and circumstances of the tumor have already sufficiently explained its character. In most cases of splenic disease, there is neither pain nor tenderness; but when the organ is inflamed, it becomes intensely tender. The tumor is the most decisive indication; and in many cases it is scarcely to be mistaken: the character is, a smooth, oblong solid tumor, felt immediately beneath the integuments, proceeding from under the ribs on the left side, a little behind the origin of the cartilages; often advancing to the mesial line in one direction, and descending to the crest of the ileum in the other; often filling the lumbar space, at its upper part. This tumor is very generally moveable; feels rounded at its posterior part; and presents an edge more or less sharp in front, where it is often notched and divided by fissures. If effusion takes place into the peritoneal cavity, a thin layer of fluid is early felt between the integuments and the tumor, but the intestines are not at any time found passing before the tumor."

It is to be regretted that the symptoms only wear a decisive character, when organic lesion has proceeded far.

The chief tumors, continues our excellent and able author, which may be mistaken for an enlarged spleen, are, chronic abscess of the integuments—scirrhous thickening of the stomach—enlargement of the left lobe of the liver—diseased omentum—feculent accumulation in the colon—diseased kidney—ovarian dropsy—hydatids.

*a. Chronic Abscess* beneath the integuments may counterfeit splenic enlargement. But, we should imagine, that no well-informed surgeon or physician, could, if careful, be misled.

*b. Scirrhous Thickening of the Stomach.*—This may occasion a tumor which, from its being obviously deeper than the integuments, and descending from below the margin of the ribs, affords the subject of doubtful diagnosis; more particularly, as scirrhus attacking the substance of the stomach, and especially the left extremity, is often quite unattended by vomiting; while, at the same time, it is apt to be attended by a sallowness of complexion, not unlike that which bespeaks splenic disease. In this case, one of the best distinctive marks will be found in the sound elicited by percussion; which, when the stomach is so diseased, is usually clear and sonorous; while the substance is still harder than the enlarged spleen.

*c. Enlargement of the Left Lobe of the Liver.*—In this case, the margin of the liver may be traced running towards the right side; while the bilious tinge of the skin and of the urine assist in the diagnosis.

*d.* The omentum may be diseased, being either corrugated into a mass, or having scirrhous or scrofulous tubercles developed in its structure. In this case, the tumor much less obviously descends from beneath the ribs—cannot be traced backwards,—extends across the abdomen—or is rough, knotted, hard, and uneven.

*e.* Feculent accumulation in the descending colon and left portion of the arch assumes nearly the situation of the enlarged spleen. The diagnosis must be sought in the history and the result of treatment. We must not, without the most persevering employment of medicines, conclude that the intestines have been emptied.

*f.* The kidney sometimes advances towards the left hypochondrium, and presents a tumor nearly in the situation of the enlarged spleen; but here we shall find, by tracing it backwards towards the loins, that its chief bulk is situated much further back, and that it is much more fixed; so that, if the patient be placed upon his hands and knees, it does not fall forwards with any freedom. On careful examination, by percussion at different times, we shall find that there is reason to conclude that the intestine lies between the tumor and the integuments of the anterior part of the abdomen when the kidney is enlarged, which is not the case with the spleen: besides all which, the history will most likely connect the tumor with some such peculiarities in the urinary secretion as will seem greatly to guide our diagnosis.

*g*. Ovarian tumors assume the greatest variety of shape. A knowledge of the fact that they may counterfeit enlarged spleen, and the general diagnosis of ovarian tumors will prevent a mistake upon this point.

*h*. The elastic feel and rounded form of hydatids will generally distinguish them from enlarged spleen, even when the situation they hold would seem to lead us to a wrong conclusion. They are, however, sometimes attached to the spleen itself.

Dr. Bright relates with more or less circumstantiality and minuteness twenty-eight cases, illustrative of the preceding observations. The extent to which we have already gone, precludes our entering on the details of these particular facts. We must refer such of our readers as would wish to see them to the volume of Reports. The few remarks with which the paper ends admit of a more special notice.

On reviewing, says Dr. Bright, the cases selected as forming a fair example of those which occur in practice where the spleen is implicated, we perceive, that, in the great majority, that organ merely partakes with others in some general state of derangement, and does not itself become a separate object of treatment. In other cases, the changes in structure are so apparently casual, as neither to be capable of detection, nor, if detected, to admit of any remedial measures. Of the few which remain, the principal diseased conditions are, the congested state of the organ, its consolidation, its inflammation, and its laceration from external violence.

Dr. Bright after apologising for the length of his communication, winds up with the following remarks:—

"I may observe generally, in reference to splenic disease, that it is probable that the spleen is greatly influenced by the derangement of many of the other organs of the body; and therefore its treatment will often depend on the regulation of their functions: for we cannot doubt, that whatever acts decidedly on the circulating system, must, in some degree influence the spleen; which obviously, from its structure and appearance, receives large quantities of blood, as subsidiary to the processes of sanguification or circulation. Still, however, it is by no means an organ easily susceptible of diseased action, and withstands the effects of injurious agencies to a very considerable extent. Probably the spleen sympathizes in a particular manner with the skin, suffering from suppressed perspiration and cold and damp applied to the surface. It also appears to be affected by certain states of atmosphere, which act as a poison upon the system, evinced particularly in countries subject to marshy exhalations. It also probably suffers from interruption in the functions of the hepatic, the renal, and the absorbent systems, as seen in the organic evidence of their diseases, and partakes of the irregular distribution of blood, caused by the diseases of the heart and arteries.

From reflecting on the frequent combinations of these and other morbid states with splenic disease, we perceive, more exactly, the mutual relations and unions existing between them; and this is always an interesting light in which to view disease. The chief points of approach or contact to which our attention is directed, are the occasional intermixture of splenic disease with disease, more or less extensive and confirmed, of the absorbent system; the depressed state of circulation occurring in severe affections of the spleen; the coincidence of splenic with hepatic disease; its connexion with derangements of the peritoneum; and some relation, though probably only collateral, between that state of the kidneys which produces albuminous urine and derangement of the spleen. By holding these and such like points in our minds, we shall comprehend, more fully, the possible value of the knowledge to be derived from following out the history of the derangements of the spleen, than we should by simply considering the morbid states of an organ of which so little is known with certainty: for the enumeration of morbid conditions can, at best, only be viewed as forming an alphabet for the construction of a language, into which we may hereafter translate the complicated and obscure legends of disease."

We think it almost unnecessary to add that we think this a highly interesting paper.

### IX. Physiological Observations on the Muscles of the Eye. By Bransby B. Cooper, F.R.S.

The precise actions and uses of the oblique muscles of the eye have been a subject of dispute and difficulty to physiologists. Mr. Cooper has made four experiments, and offered some remarks with the view of assisting in the elucidation of the subject.

A. *Superior Oblique.*—The action of this muscle is to direct the pupil downwards and outwards, and to draw the globe of the eye forwards; thus antagonizing the recti.

B. *Inferior Oblique.*—" The use of this muscle is, to direct the pupil upwards and outwards, and to assist in drawing the globe forwards. The opinions, however, which are given by different authors on the action of this muscle, vary, perhaps, more than in the description of the influence of any other muscle in the body; which circumstance may probably be attributed to a want of attention to the degree of contraction of the muscle during the period of examination; for it will be found in the dead subject, that if the inferior oblique be only slightly drawn towards its origin, the pupil will be directed upwards and outwards; but that if it be forcibly pulled, so as to equal its supposed most perfect contraction, the eye will be made to revolve on its own axis, and the pupil will become directed upwards and inwards;—an experiment which accounts, in my opinion, for the incongruity which is found in the published accounts of the influence of this muscle on the direction of the eye. It is very difficult, if not totally impossible, to judge of the action of the oblique muscles, and the effect of their contraction upon the eye, in the living animal: although most authorities agree in the accounts they give of their influence upon the organ of vision, still none have explained by what kind of experiment or observation they have arrived at their conclusions."

C. *Experiments on Rabbits.*—Before he made any actual experiments upon rabbits, Mr. Cooper first carefully dissected the muscles of the eye, and the nerves which are distributed to them in the rabbits, to ascertain if there were any anatomical reasons why an analogy should not be drawn, in the use of their muscles, with those of the human subject; and could discover none, beyond the existence of the retractor muscle of the globe, common to most quadrupeds. The straight muscles bear, in every respect, a close resemblance to those of the human subject. The superior oblique muscle, also, is very similar in its attachments and direction: arising from the posterior part of the orbit, passing forwards towards the inner angle of the eye, and there becoming tendinous, it runs through a fibro-cartilaginous ring, and is then reflected backwards, at a very acute angle; and again becoming muscular, is inserted into the sclerotic coat, beneath the superior straight muscle, and posteriorly to the great circumference of the globe.

The inferior oblique muscle in the rabbit does not bear so strong a resemblance to the corresponding muscle in the human subject as the other muscles of the organ of vision. It arises, broad and fleshy, from the most anterior internal and inferior part of the orbital cavity, takes its direction downwards along the floor of the orbit, and then passes upwards, outwards, and backwards; expanding, as it terminates, to be inserted in the outer part of the globe, posteriorly to the insertion of the recti muscles: it seems, however, that some few of its fibres pass towards the anterior part of the sclerotic coat. In proportion to the size of the globe of the eye, this muscle is larger than in the human subject, is more deeply seated within the orbit, and has a broader attachment to its inner wall.

On pulling the muscles with the blades of the forceps in the direction of their course of action, Mr. Cooper invariably found that:—

On acting on the superior oblique, the point of insertion of this muscle was drawn upwards, forwards, and inwards: the globe of the eye, at the same time, turning on its own axis, and somewhat projected forwards from the orbiter cavity, had the pupil directed downwards and outwards, corresponding with the description usually given of the natural action of this muscle.

Acting in a similar manner on the inferior oblique muscle, its point of insertion was made to roll from without inwards, and slightly from behind forwards, directing the pupil upwards and outwards. If, however, the force were still increased, but yet not apparently beyond the natural contraction of the muscle to one-third of its length, the pupil was made to roll under the eye-brow, so as to be turned inwards;—a direction, however, which is greatly increased, if the eye-lids be brought in contact, as might be felt by the globe rolling under the fingers.

Mr. Cooper having carefully dissected the muscles of the rabbit, and adopted these simple means of determining their action, next divided, with every possible precaution, the muscles in the living animal.

"The following were the results, as presented in five experiments which I performed; in each of which the superior oblique muscle was divided in the one eye, and the inferior oblique in the other. Invariably, a considerable difference is observed with respect to the degree of prominence of the two eyes: that one in which the superior oblique muscle had been divided never presented the same degree of prominence as the eye in which the inferior oblique had been cut through, seeming as if it were really preternaturally drawn into the orbit; while the eye in which the inferior oblique had been cut through looked as if projected; which, however, I think, is only attributable to the contrast between the two. The whole globe of the eye, on the side on which the superior oblique is divided, becomes depressed, as if resting on the floor of the orbit, the aspect of its cornea and iris rather dull, the pupil somewhat smaller and slightly turned inwards, and the power of vision, as far as I was capable of judging, impaired.

It is to be observed, that the retraction and depression of the globe form the principal results of the division of the superior oblique muscle. Some may be disposed to dwell on the slight direction inwards of the pupil; and contend, that the preponderating influence of the inferior oblique now produces that inversion: but it seems to me, that the combined action of the adductor and inferior oculi muscles, having lost the moderating influence of the superior oblique, would necessarily give this permanent direction to the eye; for the inferior oblique, at any rate, would have a tendency to direct the pupil upwards, whatever may be the dispute as to the direction outwards or inwards.

On the division of the inferior oblique muscle shortly after the experiment, and as soon as the animal had become quiet so as to enable us to examine the position of the eye, the globe appeared much projected from the orbit, and raised towards its roof, the pupil being directed outwards and slightly backwards. This condition of the organ followed as the result of every experiment I performed; nor did any change of position in the eyes take place, although some of the animals were kept a week or ten days after the experiment.

The power of vision seemed always most considerable in the eye in which the inferior oblique muscle had been divided, as was proved by the efforts the animal made to avoid any foreign body projected towards it: and, indeed, the manner in which it seemed capable of rolling the eye upwards and inwards, to avoid injury, leads me much to doubt the opinions of those who consider the oblique muscles as the involuntary safeguards to the eye.

The general deductions which may be drawn from the result of these experiments are, that the oblique muscles, when acting together, suspend the eye-ball in a central position in the orbiter cavity, moderate the retracting influence of the four straight muscles, and, when acting in succession, without being restricted by the influence of the recti, they roll the eye on its own axis, drawing the globe forward, and at the same time tending, in a great degree, to extend the sphere of vision. The latter use appears to me a fair inference, from the great loss of mobility the eye sustains after the division of either of the oblique muscles. In one or two of the rabbits I divided both the oblique muscles in the same eye; the result of which experiment was, the permanent retraction of the globe within the orbit, its depression on the floor of that cavity, the contraction of the pupil, without, however, any lateral direction. I am aware that the precise direction given to the pupil by the contraction of either of these muscles has not been proved by the foregoing experiments; as, upon the division of one of them, the eye is not left controlled by the action of the other alone, but must necessarily partake of the influence of the recti muscles. Much, therefore, I feel convinced, is yet left to be done by the pathologist rather than by the experimentalist."

We cordially agree with Mr. Cooper, and we think that physiology is under obligations to him for his observations.

This concludes the present number of the Reports before us. We have presented a complete epitome of its contents, and it cannot be considered as otherwise than creditable to the officers of Guy's Hospital.

---

### Hæmatology.—M. Magendie's Lectures on the Blood.

We have long entertained a great respect for the genius and talents of our Parisian confrere, M. Magendie. Ever since he cured that terrible disease, hydrophobia, by injecting warm water into the veins of a non-hydrophobic patient—and painted the theatrical portrait of cholera at Sunderland, we were convinced that the French physiologist was a genius of the first water. That terra incognita of physiologists and physicians—the Blood—is just the sphere for an esprit fort, like that of Magendie, to revel even to luxury! It is indeed—

> A wild where weeds and flowers promiscuous shoot,
> A garden tempting with forbidden fruit.

And if a Magendie does not cultivate it to perfection, he is not the man we take him for. To use another metaphor:—this terra incognita will furnish a lever by which the modern Archimedes of medicine may capsize every physiological and pathological system hitherto erected, without the trouble of constructing a new one in their place. It will prove a sea in which the Solidists, like the Hosts of Pharaoh, will be swallowed up—the humoralists made drunk—and the eclectics bewildered.

"It is surely not going too far to assert, that the manner in which we explained the origin and *mechanism*, if I may so speak, of various diseases, is of a nature to clear up, in no contemptible degree, the mysteries of pathology, of that science which cannot be said even yet to exist; for I set but little value on the minute examination of the traces left by disease on our organs, though that pursuit has been pompously styled pathological anatomy."*

Hear that ye Carswells, Hodgkins, *et hoc genus omne*, and blush for the expenses to which you have put us, and the mazes of error into which you have led us!

"Like me, Gentlemen, you have no doubt been struck with the trifling good that study confers on society. But could it be otherwise when there is scarcely a sound idea on physiology abroad?"

Mark that, ye Blumenbachs, ye *Magendies*, (of *former* days), ye Mayos, and ye Elliotsons, who have written, translated, and commentated, till the presses have groaned under the weight of your tomes! Talk of animal magnetism after the following exploits.

"You saw me give rise, at *my pleasure*, to pneumonia, scurvy, *yellow fever*, typhoid fever, &c., not to mention a number of other affections which, so to speak, I called into being before you."

M. Magendie can, of course, lay these spirits in the Red Sea, as easily as he calls them from the vasty deep. He tells us, in the same lecture, that physicians can accurately note the phenomena of diseases at the bed-side, mark their phases and prognosticate their terminations.

"Very true; but do you imagine that the nurse, provided she be habituated to her calling, does not know all that quite as well as he." The BLOOD! The BLOOD! (as our friend Dr. Wilson would say), will, in future, make the grand distinction between doctors and old women.

"All he (the physician) can do is to order certain remedies which, if necessary, the nurse could prescribe equally well."

Our highly gifted author illustrates this incapacity of medical practitioners by phthisis. "Eminent observers have described all its phenomena, even to the minutest details." Will this knowledge throw any light on its treatment, says he? "Not a particle." But the BLOOD will enable us to accomplish miracles.

"*Perhaps* tuberculous matter may be detected in the blood, and, as a further step, the means of destroying it, or preventing its formation, ascertained. Hear a fact well suited to give confidence to our hopes. We know, in the first place, that tuberculous may be accurately distinguished from purulent matter under the microscope. Now, within the last few days, in examining the body of a woman who died of phthisis, I found among the pillars of the right ventricle of the heart certain bodies resembling a sort of fibrinous sac, and containing a fluid like the pus of a phlegmonous abscess. I carried the part home and satisfied myself, by microscopical examination, that the supposed pus was, in reality, tuberculous matter; it is clear, therefore, that it existed in the blood."

There's a discovery for you! Tubercles between the pillars of the right ventricle!! This fellow will be the death of us! Is it physically impossible that tuberculous matter could be taken into the blood from depôts in the lungs?

Passing on to the second lecture of our ingenious confrere we come to a much more notable and important discovery than that of tuberculous matter in the ventricles of the heart. It is this—that blood-letting, so far from curing, is the direct cause of inflammation. The announcement is so astounding that we must quote the passage entire.

"You are already acquainted with a great number of causes that modify the blood and induce disease; but you are scarcely, perhaps, prepared for the announcement, that by means of a therapeutical agent, holding the first rank amongst the fashionable remedies of the day, I produce the very same alterations in the blood, and, as their result, the *very same disorders* in the economy. This may,

* Lancet, No. 787. p. 41.

perhaps, strike you as a random assertion, but my words are not lightly spoken. I have within my reach the guarantee of their veracity; experiment shall confirm them. I assert, then, loudly, and fear not to affirm it, that BLOOD-LETTING induces, both in the blood itself, and in our tissues, certain modifications and pathological phenomena, which resemble to a certain extent those we have seen developed in animals deprived of atmospheric oxygen, of drink and of solid food. You shall have the material proof of the fact. Here are three glasses containing blood drawn from a dog on three different occasions, at intervals of two days. The animal was in good health, and I took care to supply him abundantly with nourishment. In the first glass you see that the serum and clot are in just proportion to each other. The latter, which is perfectly coagulated, forms about four-fifths of the entire mass. This specimen of blood consequently appears to possess the desirable qualities. Now turn your attention to the second glass. The animal was still well fed when its contents were drawn, and yet you perceive an evident increase in the quantity of serum; the clot forms at the most only two-thirds of the whole. But here, in the produce of the third venesection, although the animal's diet remained unchanged, we find a still greater difference. Not only is the proportion of serum more considerable, but its colour is changed. It has acquired a reddish-yellow tinge, owing to the commencing solution of the globular substance."

See what an effulgent light is thrown on the most common matters by the fire of genius. Most people of common sense and common observation would have come to the conclusion, that if a dog, or even his master, were bled day after day, the crassamentum would decrease and the serum augment in proportion. This however is only the first step in the brilliant discovery. Let our readers pay attention to the next passage.

"I will *continue to bleed* this animal from time to time; but I can tell you beforehand, from the result of similar experiments, that the alteration in the properties of its blood will entail that of its organs, and finally death. The lungs, for example, will become affected with *engorgement, œdema,* pneumonia, and the entire train of what people are pleased to call inflammatory phenomena; and mark the extraordinary fact, that this inflammation will have been produced by the very agent which is daily used to combat it."

Now it is more than thirty years ago that Dr. Kelly, Dr. Sanders, Dr. Seeds, and others, bled animals to death, and found that while the blood-vessels, tissues, &c. were blanched, certain vital organs, especially the brain, were gorged with blood, as though Nature had attracted the remains of the vital fluid to the great citadels of life. But who would have dreamt of putting down these engorgements, watery effusions, &c. as active *inflammations*—and still less of drawing the inference that bleeding would produce, instead of curing, inflammation! Every practitioner, on this side of the Channel at least, knows that if venesection be carried beyond the salutary point in pneumonia, effusion will take place into the air-cells of the lungs or cavities of the chest. But they also know that, unless bleeding be carried to this salutary point, effusion of blood will take place, and sudden and painful death will ensue. These hæmatophobic doctrines of M. Magendie, then, are fully as pernicious as the Brunonian system developed nearly half a century ago. These lectures, delivered with all the enthusiasm of a French genius, will have a precious influence on the English students there and in Germany! Magendie tells them, bleed in health, and inflammation will be produced—*argal,* bleed in inflammation, and the disease will be augmented! These tenets will be imbibed in the lecture-room—acted on in the bed-room—and the consequences will be, that these elèves will destroy their patients, and ruin their own professional prospects! Within these few days we saw an instance where pneumonia was suffered to rise to a point that baffled all remedies, by a young practitioner deterred from the lancet by the doctrines of Magendie. It is true that our ingenious author makes a kind of salvo, by telling his audience—"do not suppose, however, that I would recommend the practice of blood-letting to be *completely* discarded." Generous latitudinarian! But will this admission avail against the dog-destroying experiments constantly before the eyes of gaping elèves, incapable of discriminating between passive congestion from watery blood, and active inflammation from rich and sizy blood? Magendie has artfully thrown in pneumonia amongst œdema and engorgement of the lungs. We take the liberty of doubting that actual *pneumonia* ever results from venesection. But even if it did take place in the case of a *healthy* animal bled into *disease and death*, would any man out of Bedlam conclude that pneumonia would be increased by venesection?

In the second lecture M. Magendie broaches another crotchet, which he carries to a most ridiculous excess. From some experiments which he has performed he concludes that alkalies—carbonate of soda, or potash, for example, are highly injurious by *thinning* the blood and inducing inflammation! It is not long since a clever practitioner in one of our medical societies inveighed against the now general use of soda, as pernicious, on the authority of Magendie.

"Now we have often had occasion to observe, that an excess of alkalinity in the blood interferes notably with the freedom of its passage through the capillary vessels. When thus modified it penetrates their walls by imbibition, is extravasated into the surrounding tissues, and, among other effects, produces in the mucous membranes the disorders long known under the name of inflammation."

Thus, because M. Magendie rendered the blood of dogs so alkaline by injection into the veins—that is, so *watery and thin*, that it would *run through the sides of the capillary vessels, but not through their patulous mouths* (!!!) we are to prohibit soda and the alkalies in dropsical effusions and other complaints, taken into the stomach, and thus acting on the kidneys, and relieving the swellings! Was there ever such wild and non-practical doctrines emitted from professor's chair in this world before? And as to the very general use of soda and potass in the present day—why are they taken? For dyspeptic complaints, where acidities are notoriously predominant in the primæ viæ—where scrofulous enlargements of the glands prevail—and where uric acid appears in the urine. In these cases the alkalies are neutralized in the stomach and bowels, and so far from proving injurious, they are actually beneficial, even by their chemical qualities. These and other portions of the lectures under consideration prove most unequivocally that M. Magendie is a mere physiological experimenter, without any knowledge of practical medicine. He tells us that nurses are as good as physicians at the bed-side of sickness—and he tells us truly, as far as he himself is concerned. In fact, we had rather trust our bodies to an experienced nurse at any time, than to M. Magendie.

*Lecture the Third.*—While we censure freely what we consider to be practical errors in the lectures of M. Magendie, we shall never withhold our assent or approbation from those portions which we can conscientiously approve. In the opening of this lecture there is a doctrine propounded which every reader of this Journal will recognize as one which we have strenuously maintained for 25 years past—namely, that the changes of structure observed, *post mortem*, in fevers, are the consequences and not the causes of the disease.

"For an example of the error into which people fell in this way, let us take the fever called typhoid. In this disease the mesenteric glands are found considerably enlarged, the intestinal mucous membrane, and especially the follicles, ulcerated, besides many other disorders. Well, they ascribe the malady to these lesions, but they are wrong. These are the *consequences* of the disease, the *anatomical proofs* that it has existed, but they are not its STARTING POINT."

In this we agree with our author—in this opinion we have long anticipated him. But we do not altogether concur with him that—"with the present mode of studying pathology that 'STARTING POINT' will never be discovered." The path which M. Magendie proposes, instead of our present mode of studying pathology, is by experiments on animals. Now, we unhesitatingly declare our solemn conviction that the present mode of observing symptoms during life, and recording the post-mortem phenomena is infinitely more likely to gain the starting-point, than that of torturing animals to death, either by food, physic, or the scalpel, and then observing the phenomena in the dead body. And here we will point out a fundamental error which vitiates every experiment that Magendie makes, and every inference that he draws. Let the practical reader reflect on the following sentiment, and ask himself whether its author has the remotest title to that of a careful clinical observer.

"For my part I declare loudly that I look upon these ideas about vitality, and the rest of it, as nothing more than a cloak for ignorance and laziness."

Thus all the phenomena observed in sickness and in health are to be explained by the laws of chemistry and mechanics! Let us just glance at some of the errors and absurdities into which this humoro-chemico-pathology will constantly lead its disciples. Dolorous mental impressions will, we know, change and derange the gastric juice, the chyme, the chyle, the bile, the urine, and, in short, every secretion that issues from every gland in the alimentary canal—and, as M. Magendie admits—will vitiate the blood itself. This will take place almost instantaneously when the individual is in good health, but crossed by grief or misfortune. Now if blood were drawn from such a person, and taken, to M. Magendie, like a bottle of urine to Dr. Laing, the chemical pathologist would pronounce the individual to be in a most desperate condition—most likely in the last stage of typhus. But, in a few days, the dolorous impressions clear away—digestion and assimilation become restored, and the blood assumes its normal condition. The blood then drawn and shewn to the professor would be pronounced the blood of a person in sound health. See then the monstrous absurdity into which the chemical pathology, in exclusion of vitality, leads M. Magendie. According to his doctrine the vitiated state of the blood must have been the *cause* and not the *effect* of the mental emotions! This comes of throwing overboard the agency of the nervous system, or, in other words, of vitality, in the disorders of digestion and the vitiation of the fluids.

After ridiculing the theories which have been broached to account for the passage of the blood from the arterial to the venous capillaries by so slight a force as the heart and vessels exert, we are dazzled by the following effulgent explanation.

"My inquiries up to the present time, go to prove, that the passage of the blood from the arterial to the venous capillaries, is effected by means of the nice adaptation of its *physical properties to the physico-vital endowments of the vessels.*"

Most brilliant elucidation! The light which is here thrown on the subject is too bright for human eye to bear—and consequently we are just as much in the dark as ever.

If we can understand our lecturer, he conceives that the passage of the blood through the capillaries depends not on the liquid but coagulable fibrine contained in it; for if alkalies be injected into the veins of an animal, the coagulability of the fibrine is lost—the blood stagnates in the capillaries—and congestion, effusions, or even inflammation ensues. This, he affirms to be the case in most of the epidemics, and especially in the late one called the "*grippe.*" Without questioning these assertions or opinions, we will now extract a passage which will make every practical man stare.

"Again, *in acute rheumatism,* as I once before explained to you, the painful parts become the seat of engorgement due to the stoppage and accumulation of the blood in its canals. The liquid stagnates, *its temperature falls,* and hence the sensation *of cold felt by the patient, and which may, in some cases be felt by a bystander on the application of the hand.*"

Have we practised for forty years, and yet were so blind and unobservant as not to perceive that the joints in *acute rheumatism* felt cold, even to hands of bye-standers! If the Seine is ever to be set on fire by mortal hand, M. Magendie will be the man.

M. Magendie has found or fancied (which is the same thing) that acetate of ammonia facilitates the passage of blood through the capillaries, and avers that he cures his rheumatic patients by this remedy alone. He says he has never lost a single patient in acute rheumatism, either in his hospital or in private practice —and we can well believe him—if they presented *cold joints* in this painful disease. The following sentence is praiseworthy—and it is more applicable than the author means it to be.

"All this is accurately true; nevertheless, did I assert it in too exclusive a manner, I should, in my turn, be guilty of an error. Nothing is so easy as to deceive oneself into the belief of the universal applicability of any important fact one may be fortunate enough to discover. Against such self-deception it is my duty to guard you."

We doubt if there is a single spot on the surface of civilized Europe, except Paris, where a medical audience would listen to the absurdities broached in Magendie's lectures, without bursting into laughter, or deserting the room.

We have already alluded to M. Magendie's crotchets respecting soda, and its properties of causing inflammation, by making the blood so thin as to run through the sides of the arteries, but incapable of running through their patulous mouths into the venous capillaries. Listen, readers, to the proofs which he adduces of this important doctrine.

"My experiments have demonstrated the property possessed by this body of liquefying the blood, by combining with its fibrine. Now, I have not a doubt but that its long-continued medicinal use produces similar results on the blood, causes infiltration of the lungs, and acts, in short, so long as persisted in, as an inexhaustible source of pneumonia; at least, I am authorised in this belief by what occurred to a friend of mine, one of the most celebrated men of the present day. He has been compelled to give up taking the carbonate by a succession of pneumonic attacks, that followed each other while he was in the habit of employing it."

Thus, because a friend of M. Magendie's happened to have a series of pulmonic attacks, while using carbonate of soda, these attacks were the consequence of its use! And such reasoning is issued and swallowed in the year 1838! Alas for physic! No wonder that our profession is becoming the laughing-stock of the scientific and rational world, when animal magnetisers and hæmato-maniacs are listened to by men professing to have brains in their skulls!

*Lecture* 4.—This lecture commences with a kind of determination to throw overboard sensibility, contractility, and other vital phenomena in the circulation, and to adopt physics and hydraulics in their stead.

"There is a fact, in physics, remarkable for the excellent term of comparison which it serves to establish between the phenomena of the movement of the blood in our organs and the circulation of liquids in inert tubes. I allude to the enormous pressure which is required in order to make water pass through a tube of very small diameter, while the blood traverses with ease the infinitely more minute tubes that abound in our tissues. There must be some particular conditions to facilitate its passage. What proves their existence is, that if certain alterations are effected in the composition of the blood, it stops, undergoes morbid changes, becomes extravasated and decomposed, and produces the various disorders which pathologists have vainly attempted to explain by the words *inflammation* and *irritation*. *What sense, in truth, is there in applying the words inflammation to our organs? Do our tissues really take fire? I confess I know of no example of such a phenomenon.*"

Thus M. Magendie's assumptions that the capillary vessels have nothing to do with the capillary circulation, and that the passage of the blood through them depends entirely on certain qualities of the fluid itself, are perfectly gratuitous—and unsupported by facts or reasoning. Upon the principles of physics and hydraulics, how will he account for the crimson cheek, in shame, while no other part of the surface turns red? Has the heart power to direct more blood to the capillaries of the face than to any other capillaries? The idea is absurd. He tells us, in direct words, that the vessels do not possess contractility!

"The questions we have so far treated of belong, in great measure, whatever may be said to the contrary, to the subjects of physics and hydraulics. You will find that, in elucidating them, we shall have *no occasion to avail ourselves of the so-called contractility of the vessels, which, in reality, does not exist*; and we shall be quite as independent of the species of *sensibility*, termed *insensible*, and of the entire collection of absurd terms by the help of which no few romances, doubtless very clever, but very empty too, have been got up about the capillary circulation."

As to his objection to the term "INFLAMMATION," it is the most puerile effort at wit which we ever remember to have seen. Because the great toe of a man in gout does not flare up, like a gas-lamp, the term "INFLAMMATION" is ridiculous!

Against "irritation" too, our author wages war to the knife.

"What on earth is the meaning of the word *irritation*? An obstacle of some sort or other modifies the course of the blood in any given organ, and instead of simply stating that there is a modification produced by a mechanical impediment to the progress of that fluid, you tell us the part is *irritated*, and actually employ the very term the worst fitted to designate the disorder manifested in the circulation How long, I should be glad to know, have our organs been proved susceptible of feeling passions—of becoming irritated—I had almost said of getting angry?"

The only comment we shall make on such exquisite absurdities shall be in the words of Magendie himself.

"The verbiage and subtlety of the bar seem to have spread to the members of our profession. Whoever talks loudest and longest usually triumphs, and, of course, *settles the matter his own way: he does so often in defiance of truth*; but what matters for that? he gets himself spoken of and makes a name. On the other hand, the man who does not possess that extraordinary gift of the tongue, by which white is made black and black white, remains unknown, vegetates, and passes for an ignoramus in the eyes of the public."

We should be inclined to laugh heartily at the new-fangled doctrines of the profession, did we not know that no doctrine or practice can be so preposterous or pernicious as not to entrap a considerable number of medical practitioners, and especially of medical students. We must therefore endeavour to check the evils which M. Magendie's lectures are calculated to disseminate through society. What will the *practitioner* say to the following passage?

"We were speaking of the viscidity requisite for the circulation of the blood through our organs. Now here is the blood of an individual who had an attack of hemoptysis, and was bled freely for it. You know well what I think of that remedy,—worse, perhaps, than the disease. Be that as it will, you may perceive that this blood is very slightly viscous; I, in consequence, presume that further mischief will occur."

Heaven help the unfortunate hæmoptisical patients who may come under the care of this visionary enthusiast, whom experience (if he really has any) must have blinded rather than enlightened! We are almost weary of exposing the matchless absurdities that crowd every page of these lectures; yet still we must proceed a little farther. Who, out of Bedlam, would compare the effects resulting from injections into the veins with ingurgitation into the stomach?

None but Magendie. He injects mucilage of gum-arabic into the veins of an animal till the vessels are choaked up and the animal dies, and he does not appear to think that there is the slightest difference between this process and that of taking gum-arabic into the stomach, where it has to undergo *digestion* and assimilation before it enters the circulation!!

"You remember the experiments we made to exemplify this question. We added a viscid matter, innocuous in itself—gum, for example,—to some water, and, after colouring the fluid, injected it into the jugular vein of an animal. So long as the injection traversed the large venous trunks no disorder was occasioned; but once it had arrived through the pulmonary artery, amongst the minute ramusculi of the lung, its degree of viscidity ceased to be in just proportion to the capacity of the tubes. The consequence was, that the circulation almost instantly stopped, and, as the encephalon no longer received the necessary excitement of arterial blood, its functions ceased, and the animal quickly perished. The autopsy was immediately made, and, on incising the pulmonary parenchyma perpendicularly to the direction of its principal vessels, we invariably found them stuffed with the substance injected. Let us admit, however, that this liquid had succeeded in making its way through the capillary system of the lung. You are aware that the diameter of the ultimate tubes varies almost in every organ, and that in some of them it is still smaller than in the lungs. Let us suppose, then, any substance that has passed through the pulmonary parenchyma with great difficulty, arriving at other capillaries of greater tenuity. It will, beyond question, be arrested in its course by this new obstacle; and its stagnation and subsequent effusion will produce, according to the nature of the parts with which it is in contact, various disorders, more or less analogous to those already described."

*Lecture* V. In this lecture, after slaying, for the tenth time, the doctrines of vitality, irritability, &c. our Professor rises into the miraculous, and assumes the attributes of a modern Prometheus.

"But, Gentlemen, it has been shown to *demonstration*, that the empire of inflammation is even more widely extended than it was the habit to assume; and, what is remarkable, *it is I who, like a generous enemy, have conferred on it powers of which even its very warmest partisans had deprived it. They* limited its action to living organs; *I* have extended it to the tissues when they have ceased to live. Many and many a time have I proved, by experiment, that its most terrible symptoms develop themselves in parts *wholly inanimate*."

The animal magnetisers may now hide their *diminished* heads—if they have any heads left—and the Okeys may go back to their old occupations in the garret.

No man can, with more adroitness than M. Magendie, dilute a small quantum of truth with a large mixture of misrepresentation, when an opponent is to be turned into ridicule. Yet no process is more dangerous, or more dishonorable. Thus, after giving a short, and by no means graphic sketch of a chlorotic girl, M. Magendie observes—"in order to remedy these *organic disorders* (what a phrase!) the practitioner *bleeds* the patient, and gives preparations of iron." Now if the professor were closely questioned as to the practice of *bleeding* in chlorosis—*merely as chlorosis*—he would probably reply that he knew not whether such was the practice—nor did he care. His object was to produce a "sensation" amongst his elèves, no matter at what expense of his professional brethren!

The beautiful and accurate deductions of Laennec and all our modern auscultators are thrown overboard, and from some squirting experiments in leathern and metallic tubes, M. Magendie boldly avers that the "bruit de soufflet," "bruit de scie," "bruit de diable," &c. have nothing to do with affections of the heart itself, but merely with the composition of the blood! With a man who outrages reason—denies facts—and maintains fictions—it is useless to argue.

We shall not, therefore, pursue these lectures any further *at present.* We have pointed out sufficient samples of their unparalleled absurdity—their shameless illiberality—their unsparing criminations of the profession at large—and, worse than all, their most pernicious tendency. Let the medical students, and the junior practitioners beware how they embrace M. Magendie's tenets, or act on his principles. If they do not, they will sacrifice their own reputations—and, what is of more consequence, the lives of their patients.

---

An Introductory Discourse on the Studies required for the Medical Profession. Addressed to the Students of the Medical School of St. George's Hospital, October 1, 1838. By Sir Benjamin C. Brodie, Bart. F.R.S. Serjeant-Surgeon to the Queen, and Surgeon to St. George's Hospital.

This Discourse was delivered in the School of the Hospital, to a crowded audience, at the commencement of the present session. Sir Benjamin Brodie has not only attained the highest place amongst existing British surgeons, but he has fully deserved it. His early diligence has been equalled by his subsequent industry, and the acuteness of observation and soundness of judgment, which form the characteristics of his mind, have struck all who have perused his writings, or enjoyed the honour of his acquaintance. A discourse on medical study, from him, must command attention and respect. The old may compare his experience with their own—the young may learn the road to success from one who has himself trod it.

We shall extract such passages from this Address, as are calculated to be useful. They are far from few. Sir Benjamin is essentially a practical man, and his strong sense detects and exposes the fallacies and sophisms of the day.

1. Speaking of the *Treatment* of Disease, Sir Benjamin observes :—

"Let it always be borne in mind that this last is the real object which you have in view. I address you as future medical practitioners. If, taking another course, you choose to study anatomy and physiology, merely as interesting branches of human knowledge, you are at liberty to do so, and you will be as well rewarded for your labours as if you had applied yourselves to geology, optics, or astronomy. In like manner, if any one apply himself, as a philosopher, altogether to the study of pathology, he will find much in it that may interest himself, and that may be useful afterwards to those who carry their researches further. But as medical practitioners, you must not stop at either one or the other of these points; and, never losing sight of the ultimate object of all your investigations, you must estimate the value of whatever other knowledge you acquire by the degree in which you find it to be directly or indirectly applicable to the healing art.

It is one advantage arising from the peculiar constitution of the London medical schools, that, with few exceptions, the instructions, which you here receive, have, in a greater or less degree, a tendency to practice. The ambition of the teacher of anatomy is not limited to success in his present vocation. He looks forward to the time when his profession as a physician or surgeon will elevate him to fame and fortune. His mind is naturally directed to those inquiries, a proficiency in which will most assist him in the attainment of these objects; and that which is useful to himself cannot fail to be useful to his pupils. I have no doubt that the praises which are bestowed on some of the continental anatomists are well founded: that there are universities in which the anatomical professors, devoting their whole time, and industry, and intellect, to this one pursuit, explain the mysteries of minute anatomy at greater length, and with more precision, than the teachers here: but, nevertheless, I assert that ours is the better method with a view to the education of those who wish to become, not mere philosophers, but skilful and useful practitioners.

In like manner, pathology is not taught here as a separate science, but you receive your instructions in it from the lecturers on the practice of physic and surgery; who, while they explain the changes of function or structure, which constitute disease, point out also the symptoms by which the existence of these changes is indicated in the living body, and the means to be employed for the patients' relief. Thus, while you are taught pathology, you are taught also its uses and application; and these different subjects, brought under your view at the same time, serve mutually to elucidate each other; for, while pathology assists you in obtaining a knowledge of symptoms, the study of symptoms, and of the operation of remedies, contributes in no small degree to extend your knowledge of pathology."

There is much in these remarks that calls for serious consideration. Medicine is partly made up of abstract, partly of practical science. As philosophers, we may cultivate the former—as physicians and surgeons we must apply, and should be familiar with, the latter. The mass of students are neither calculated nor intended for mere speculative men. They require that practical knowledge, without which they can neither feel nor inspire confidence, and which is indispensable for the safety of their patients, and the success of themselves.

But latterly a disposition has been shewn in some of the London schools, to erect separate chairs of physiology and pathology, and to teach them in their transcendental form—a plan which will probably be attended with ultimate mischief to the pupils, and transform them into theorists, instead of plain and sensible practitioners.

2. It would be well if students would take this hint:—

"It is of great importance that you should so arrange your studies that no excessive and overpowering demand may be made on your attention at any one period. And here let me advise you to begin with a system of steady application, and to adhere to it throughout. It is not uncommon for medical students, any more than it is for other students, to engage at first with zeal in their pursuits; then, as these lose the charm of novelty, to become careless and indifferent, and, at last, when their education is drawing to a close, and it becomes a question how far they are qualified to undergo the required examinations, to endeavour to make up for the time that has been mis-spent and wasted by excessive labour, such as is incompatible with sufficient physical repose and mental relaxation. But it is not in this way that great things are to be accomplished either in our profession, or in any other. Habits of attention which are once lost are not easily regained; and no durable impressions are made upon a mind which is exercised beyond its powers."

3. Sir B. Brodie recommends devotion of the first season to anatomy. If the student has five or six years before him, he should dedicate two Winters to this department of science, before he attends the hospital.

"While engaged in attendance on the hospital, always bear in mind that there is no one of your other studies which, as to real importance, can compete with this. The lectures on anatomy, chemistry, materia medica, practice of medicine and surgery, and midwifery, are nothing in themselves. They are but the means to an end, and are valuable only because without them you would be unable to learn the symptoms and treatment of diseases in the hospital. I feel it my duty to make this observation, and to make it earnestly, because it appears to me that the truth which it inculcates is not, for the most part, sufficiently impressed on the minds of medical students. Perhaps, however, if strict justice were done to all concerned, and we were to trace this mistake to its origin, we should find that it belongs, not so much to the medical students themselves, as to those by whom their course of education is regulated, and who, by a false estimate of the importance of lectures, and an unnecessary multiplication of the number of them which the students are required to attend, have left an altogether insufficient time for a profitable attendance on the hospital."

Probably the Apothecaries' Company require attendance on too many lectures upon Botany, and certainly on too many upon Medical Jurisprudence. In other respects, it would not be easy to find much fault with their arrangements. The fruits of their plans may be seen in the superior intelligence of the present over the former race of professional men. We allude, of course, to the mass. The character of Ollapod in Tristram Shandy would fit few now.

4. *Hospital Attendance.*—All that Sir Benjamin says upon this subject is excellent.

" It is not by going through the form of walking round the wards daily with the physician and surgeon that you will be enabled to avail yourselves of the opportunities of obtaining knowledge which the hospital affords. You should investigate cases for yourselves; you should converse on them with each other; you should take written notes of them in the morning, which you may transcribe in the evening; and in doing so you should make even what are regarded as the more trifling cases the subject of reflection. Some individuals are more, and others are less, endowed by nature with the power of reflection; but there are none in whom this faculty may not be improved by exercise, and whoever neglects it is unfitted for the medical profession.

You will at once be sensible of the great advantage arising from your written notes of cases. But that advantage is not limited to the period of your education. Hereafter, when these faithful records of your experience have accumulated, you will find them to be an important help in your practice; when years have rolled over you, and the multitude of intervening events has obscured the once bright inscriptions on your memory.

Feeling as I do how essential it is, both to yourselves and to the public, that your hospital studies should be well conducted, I shall proceed to offer some further observations on this subject.

In the first instance, your attention should be directed more to the symptoms and progress of diseases than to their treatment. You should begin with those of the simplest form, as the only means of obtaining that elementary knowledge, without which you will in vain endeavour to comprehend the more complicated and difficult cases. Carrying with you into the wards of the hospital the knowledge which you have acquired in the dissecting-room, you will, in each individual case, make these inquiries:—What is the nature of the disease, considering it anatomically and physiologically, and in what organ is it situated, or has it no distinct locality? If these points can be satisfactorily determined, you will, in most instances at least, have discovered the bond of connexion between the various symptoms; your subsequent investigation of the case will be rendered more simple; and you will be enabled to form a more distinct and rational notion as to the treatment which is required, and the probability of a cure, than you could have formed otherwise. Do not be satisfied with having learned the structure and functions of the body in health, but attend the examination of those who have died of their complaints; and endeavour to associate the symptoms which existed before death with the morbid appearances observed afterwards. The more extended cultivation of morbid anatomy is one of the most peculiar features of modern times. It has laid the foundation of a more accurate system of pathology than that which existed formerly, and has led to many improvements in practice; and it is right that your minds should be impressed with a just sense of its great value and importance."

5. *Morbid Anatomy not Pathology.*—Sir Benjamin makes some admirable remarks upon this head.

" Morbid anatomy is not pathology, though it is an essential part of it. You may know all that is to be known of the former, and yet your knowledge of the latter may be very limited. To be a pathologist you must study disease in the living body, even more than in the dead. Even in the instance of what we call local diseases, morbid anatomy does not teach us all that we ought to know; but there are many diseases which, as far as we can see, have no absolute locality; and what does it teach us there? In cases of hysteria, gout, fever, and in a number of others, which it would be easy to enumerate, the dissection of the dead body furnishes us with little else than negative information; and in some cases, if we trust implicitly to it, morbid anatomy will prove a deceitful guide. Thus, in a patient who has died of continued fever, you find the mucous membrane and glands of the lower portion of the small intestine ulcerated. Your first impression might be that you had discovered the original malady of which the fever was symptomatic. It is only by the investigation of the disease in the living person that you are enabled to satisfy yourselves that the ulcers were the consequence, and not the cause, of the fever. The mere morbid anatomist may suppose that in the inflammation of the œsophagus and trachea, he has discovered the essence and real seat of hydrophobia; but a more extended observation teaches you that such inflammation is but a contingency; and that, whether it exist in a greater or less degree, there will be the same fatal termination of the patient's sufferings. Then there is an extensive class of diseases in which we may say that there is actually no danger; and of these morbid anatomy can teach us nothing, although we may learn much respecting them, so as to understand their nature sufficiently well, by investigating them in other ways. We know as much of a sick headache as of pulmonary consumption; as much of psoriasis and lepra, as of small-pox and measles."

6. *A Sanguine Temperament the best.*—"The first question, then, which should present itself to you in the management of a particular case is this:—Is the disease one of which the patient may recover, or is it not? There are, indeed, too many cases in which the patient's condition is so manifestly hopeless, that it is impossible for you to overlook it. Let me, however, caution you that you do not, in any instance, arrive too hastily at this conclusion. Our knowledge is not so absolute and certain as to prevent even well-informed persons being occasionally mistaken on this point. This is true, especially with respect to the affections of internal organs. Individuals have been restored to health who were supposed to be dying of disease in the lungs or mesenteric glands. But it is also true, though to a less extent, with respect to diseases of parts which are situated externally. I know females who are now alive and well, who were supposed to labour under malignant disease of the uterus; and I could mention many cases in which patients have recovered of what had been regarded as an incurable disease of a joint. It is a good rule in the practice of our art, as in the common affairs of life, for us to look on the favourable side of the question, as far as we can, consistently with reason, do so. A sanguine mind, tempered by a good judgment, is the best for a medical practitioner. Those who from physical causes or habit are of a desponding character, will sometimes abandon a patient to a speedy death, whom another would have preserved altogether or for a considerable time."

Sir Benjamin's observations on the principles which should guide us in the treatment of disease are both philosophical and practical. We wish that our space permitted their insertion. But we cannot refrain from quoting the following observations:—

"So far the rules of practice seem to be sufficiently intelligible. But the great difficulty remains to be noticed:—How are you to determine what are remedies, and what are not, and the real value of the remedies which you possess? Here is the most abundant source of the errors which infest our art; from which even the most experienced and discerning practitioners are not altogether exempt; but which especially prevail among those who are deficient in experience or good sense. It is to the almost entire ignorance of the public, and especially of the aristocratic classes, as to the evidence which is necessary to establish the efficacy or inefficacy of a particular mode of treatment, that we are to attribute the reputation which is frequently obtained by empirics and other adventurers, who pretend to practise the art, without having learned the science, of medicine.

When the optician, in constructing an optical instrument, arranges his lenses and reflectors in a new order, his knowledge of the principles of optics enables him to predict the effect which will be produced, so that, except as to some minor circumstances, he can be scarcely said to be making an experiment. But there is no reason to believe that in the study of those varied and complicated phænomena, which are the subject of Physiology and Pathology, we shall ever arrive at that point which has been long since attained in Optics, and some other branches of Natural Philosophy; and at all events, we are far distant from it at the present moment. Few greater benefits have been conferred on mankind than that, for which we are indebted to Ambrose Parey—the application of a ligature to a bleeding artery: but no knowledge which he possessed would have enabled him to say more than that it would be probably successful; and it was left for after-ages to demonstrate the principle on which it acts, and to explain the circumstances which may cause its failure. John Hunter, as you will hereafter learn, was led by his knowledge of the animal economy to propose a new method of treating aneurysm; and it is impossible to estimate the number of lives which have been preserved by this discovery; yet it was but an experiment, of which even his philosophic mind could not, with certainty, predict the result. It must, however, be admitted that science pointed out the road to these inventions. But this cannot be said of the great majority of the remedies which you will see employed. Nothing that could be known beforehand would lead you to expect that Ipecacuanha would operate as an emetic; or that Opium would occasion sleep; that Quinine or Arsenic would cure the ague; that Inflammation of the Iris would yield to Mercury; or the gout to Colchicum. The invention of these, and of a multitude of other remedies, is of accidental origin; we are indebted for our knowledge of them, for the most part, to the observations of ignorant persons, accumulated during a long series of ages; and the office of men of science is little else than to study their effects minutely, and to learn the right application of them. But even in doing this, the greatest caution and, I may say, scepticism is necessary to prevent you being continually guilty of mistakes. I have already told you how many diseases, if left to themselves, admit of a spontaneous cure. We see the surface of the body, and we know by certain outward signs a good deal of what takes place within; but there is much of which we know nothing, so that it is impossible for us to take cognizance of all the circumstances which may occur to modify the course, and alter the termination of a disease. If we trust implicitly to the instinct which inclines us to believe that when one event follows another, the first is the cause, and the second the effect, we shall be frequently directed wrong. The fact of a patient having recovered under a particular mode of treatment, goes but a little way towards establishing its value; nor is anything sufficient for this purpose, short of the same result being obtained in many similar cases, in which there was otherwise little prospect of recovery. It is the disposition of every one of us to admit the efficacy of the remedies which we employ on insufficient evidence; and unless we, whose duty it is to understand these subjects, are on our guard against this not unnatural prejudice, we have little right to blame the credulity of those whose minds are not turned to these inquiries, when a corresponding error of judgment leads them to believe in the absurdities of metallic tractors, animal magnetism, and homœopathy?"

Sir Benjamin Brodie concludes with some observations which the experience of most men confirms:—

" Although many years have since elapsed, it seems to me but as yesterday, when I was, as you are now, a young adventurer in this great Metropolis; and I well remember how often, in the intervals of my occupations, I have contemplated, with something like dismay, the prospect which lay before me. My own feelings, at that time, explain to me what may possibly be yours at the present period. Yet you have undertaken nothing which energy and perseverance, and upright and honorable conduct, will not enable you to accomplish. It cannot, indeed, be predicated of any individual to what exact extent he may attain professional success, for that must depend partly on his physical powers, partly on the situation in which he is placed, and on other contingencies: but having had no small experience in the history of those who have been medical students, I venture to assert that no one who uses the means proper for the purpose, will fail to succeed sufficiently to gratify a reasonable ambition. You have entered on pursuits of the highest interest, in which you will have the no small satisfaction of knowing that you never acquire any real advantage for yourselves which is not the consequence of your having benefited others. It is true that you have years of constant exertion before you; but you will eventually learn how preferable such a situation is to that of those individuals who, born to what are called the advantages of fortune, but neglecting the duties of their station, believe that they can direct their minds to no more worthy object than the multiplication of their selfish enjoyments. It will not be your lot, as it is often theirs, to suffer the miseries of ennui, or to be satiated and disappointed with life at an early period; nor will you have to regret, as you advance in age, that you have lived unprofitable members of society."

The lecture will be read with as much pleasure and as much instruction, as were experienced by those who heard it. The good sense and good feeling of its author are exhibited in every page, and the student who lays some of its lessons to heart, will never repent his perusal of it.

---

A CLINICAL LECTURE ON THE PRIMARY TREATMENT OF INJURIES, DELIVERED AT THE NEW YORK HOSPITAL, NOV. 22, 1837. BY ALEXANDER H. STEVENS, M.D.

This is a well conceived and well-written lecture. It takes up the subject of "shock" or "constitutional irritation after injuries," examines it methodically, and dwells on the treatment of the particular symptoms or phenomena which characterise it.

There is a question which has long been agitated and is not yet satisfactorily settled. Should a person labouring under the shock of an injury be bled? The arguments for bleeding are thus stated by Boyer in his Chapter on Injuries of the Head.

" What we have chiefly to fear after a violent percussion of the head are, sanguineous congestion, rupture of vessels, extravasation of blood, and inflammation. The most powerful means of preventing these consequences is to diminish the quantity of blood by bleeding. At the moment, therefore, when we are called to a person who has fallen or received a blow upon his head, and who has symptoms of concussion, we should bleed largely from the arm, and repeat the operation several times within twenty-four hours. If the symptoms are not relieved, a vein in the foot, or even the jugular vein should be opened. At the same time leeches should be applied to the temples. It is impossible to say how much blood should be taken away in such a case. Few cases require so large and repeated bleedings as injuries of the head. Experience has shown that bleeding is the most efficacious remedy that can be employed, and the writings of the best observers abound with cases illustrating its advantages. All writers are not however agreed as to the propriety of bleeding in the cases in question. Some looking only to the loss of the elasticity of the brain, and the stupor resulting from it, consider bleeding injurious, and prefer the use of remedies calculated to relieve the brain from its torpid state. But experience has shown that when the injury has not been severe enough to destroy entirely the functions of the brain, and to cause sudden death, there is no further danger except from extravasation of blood, inflammation and suppuration. Now bleeding is the best remedy to prevent these consequences. The number of bleedings, the quantity to be drawn, and the part from which it is to be taken, must be determined by the ability of the patient to bear them, his strength and temperament.'

In the same chapter, Boyer says, emetics are sometimes useful but generally dangerous remedies. He advises purgatives, 'warm drinks, even cordials, when the stupor continues and the weakness is very great.'

'The difficulty of treating these cases properly, arises from the uncertainty of the diagnosis. In simple stupor of the brain, exciting remedies are indicated; in extravasation they are injurious. The indications are clear when the nature of the case can be ascertained—the obscurity is in the symptoms by which the condition of the brain is manifested.'—*Boyer* Traité des Maladies Chirurgicales. Vol. 4th."

In the first place we may remark that these observations of Boyer's apply to injuries of the head. They do not refer to cases of shock in general. What weight they may have must therefore be materially diminished in the case of simple shock.

Dr. Stevens remarks :—

"I have seen many recoveries from a state of extreme prostration where either nothing was done except leaving the patient quiet, or where rest was combined with stimulating remedies; and having carefully compared the result of this treatment with that of an opposite character, I confidently recommend it to you in all cases of injury attended with great prostration, except internal hemorrhages; as in the head from apoplexy or injury, and in any of the cavities of the chest or abdomen from wounds. In these cases it is better to incur the dangers of extreme depression of the powers of life, while a clot is forming at the mouths of the bleeding vessels, than to re-excite the bleeding by giving force to the action of the heart and arteries. These remarks are especially applicable to gun-shot wounds of the lungs."

It appears to us that nothing can be more absurd than to bleed a man furiously again and again, merely because he has been knocked down or run over. Yet in a case of severe shock, particularly in injuries of the head, the consequent reaction is so likely to be severe, that a moderate abstraction of blood is not objectionable in theory, nor, we think, in practice. We say a moderate abstraction of blood, for we do consider that recommended by Boyer, decidedly immoderate.

In a slight case of concussion we have always found the do-nothing system answer very well. Quiet, cold lotions to the head, and purgatives, have answered very well in most cases. But in severe concussion, if the symptoms do not yield, those of compression or inflammatory action must be apprehended, and depletion ought to be resorted to.

Dr. Stevens notices particular symptoms. We shall insert his observations on a few.

*Jactitation.*—This occurs in various degrees as a symptom of fever of two forms: *First,* the fever with strong, full pulse, moderately accelerated. *Second,* with a very rapid thrilling pulse, easily compressed. Bleeding is applicable to the former state; the latter demands the application of cold to the head, tepid ablutions to the extremities, opium and extreme quiet of mind and body. But the sort of jactitation more immediately under consideration, occurs in connexion with coldness of the surface, a lustreless eye, and disturbed sensorium; this is a common effect of hemorrhage. Warm stimulants, external heat, sinapisms to the epigastrium, together with mechanical confinement in a draft of cool air, and frequent sponging of the face with cold water, are here indicated. When the skin has become hot, sponging with cold water, or spirits and water, or ice to the head is also useful. In many instances, confinement by bandages, pressure upon the knees so as to keep the legs extended, and at rest, will induce sleep; even the weight of a pillow in slight cases is attended with benefit. Of the internal remedies more particularly indicated by this symptom, opium and camphor stand in the foremost rank.

*Vomiting in the Intemperate.*—" Sometimes vomiting or retching occurs *in those habituated to spirituous liquors,* from withholding the accustomed stimulus: here the customary dram is required. In all cases where it continues after the stomach is emptied, it should be met by sinapisms to the epigastrium, or to the whole abdomen, applied hot, and not so strong as not to be tolerated for two or three hours. If the surgeon be not attentive and rigid in the enforcement of the proper administration of remedies, vomiting will be reinduced by an undue quantity of stimulants, or of the vehicles in which they are administered. On this point it is difficult to fix upon doses and quantities that will not require great variation in particular cases. Yet as a medium dose that can be administered in any case, I would state about two quarts in twenty-four hours: and of brandy half a pint; carbonate of ammonia one dram; the vehicle two quarts. As regards the intervals of exhibiting these remedies, once in fifteen minutes is sufficiently often for the worst period, and the quantity half an ounce. After the patient has rallied, he will be more benefited by giving larger quantities at longer intervals, and permitting him to sleep. Alternations of nourishment and sleep, are Nature's best restoratives. If you have a very careful and intelligent assistant at the bedside, the patient may be suffered to sleep until the pulse begins to flag; but after injuries, as in low fevers, patients may sleep away their strength, and relapse into coma and prostration, for want of due stimulation. The timidity of nurses not unfrequently leaves patients to sink for want of stimulus, when in fact they are able to swallow. The surgeon placing himself at the patient's right side, should gently elevate his head with the left hand, and rubbing the half-filled spoon against the lower lip of the patient, endeavour to arouse him by a decided exhortation to swallow; and at the first indication of consciousness he should pour the liquid on the back part of the tongue, and wait a few moments to see if it is swallowed, which he will know by seeing the larynx to be slightly elevated. If this does not take place, these efforts should be repeated, and the patient be again exhorted to swallow. If the attempt does not succeed, the liquid should be permitted to run out of the mouth, otherwise it may produce strangulation."

*Stimulants ought not generally to be given long after a rigor has set in.*—When a person, says Dr. Stevens, has been travelling with the thermometer at zero, and approaches the bar-room fire, he shakes and trembles, and feels cold, although he may have been comfortable on the road. The rigor is the harbinger of returning warmth. If he sits by a hot fire and takes stimulants, brandy in lieu of warm tea, a feverish condition of the system is induced. It is in this way that colds are taken. Therefore, we should be careful, lest the operation of the stimulants be carried into the state of excitement, and cause undue rapidity of circulation, and undue determinations of blood to particular organs. The serous tissues of the head, the mucous membranes of the alimentary canal, and of the lungs, are most often the seat of such local determinations.

Another bad effect of giving too much stimulus is, that vomiting is excited. "Ah, Doctor," said a shrewd bye-stander, when a medical man attempted to bleed, and the blood would not flow, " nature knows more than the physician." So she does, also, when she rejects by vomiting, the excessive doses to which the stomach is often subjected.

A third evil consequence of excessive stimulation is, that it exhausts the excitability and leaves the patient in a state of depression very difficult to manage. To stop with the stimulants is difficult; to go on with them impossible. The giving of powerful stimulants, is like borrowing money at high interest; it may do in a special emergency, but if long continued, it is sure to be followed by loss of excitability, which is the capital by which life is supported.

This lecture contains many excellent observations.

---

### New York Hospital.

Lectures on Lithotomy, delivered at the New York Hospital, December, 1837. By Alex. H. Stevens, M.D. Surgeon of the New York Hospital, and Emeritus Professor of Clinical Surgery. 8vo. Stitched, pp. 93. New York, 1838.

We shall extract a few passages from this Lecture. Dr. Stevens appears to be a zealous and an able surgeon.

1. *On the Causes of Death from Lithotomy.*

After quoting the observation of Dupuytren, that " about three-fifths die of inflammation ; the most frequent seat of which is the bladder, the cellular tissue of the pelvis, the rectum, the peritonæum, the kidneys, the lungs, the pleura, or the liver."

" About one fourth die of hemorrhage, or the means used to arrest it ; of the rest some perish from accidental or concomitant diseases : as verminous affections, measles, convulsions, small-pox, disorders of the digestive system, rheumatism, catarrhs, &c."—Dr. Stevens adds :—

" Without sufficient personal experience to offer an opinion on these most interesting and important statistical statements, I would yet remark that too little stress appears to me to be laid upon those complications of affections of the kidneys, which are found upon dissection to co-exist with stone in the bladder, and render operations improper and fatal ; and secondly, that no mention is made of those nervous symptoms, the sinking and prostration, from which it is not unusual for patients to perish ; and finally, nothing is said of phlebitis, or of diffuse inflammation, depending either upon infection, or a disordered state of the constitution."

We quite agree with Dr. Stevens in the preceding observations. We are sure that the state of the kidneys is insufficiently ascertained or weighed, previously to the performance of the operation of lithotomy.

Dr. Stevens goes on to remark that, in post-mortem examinations we find, according to Dupuytren, the neck of the bladder, and the left half of the prostate gland, freely and smoothly divided by the cutting instrument as far as the cellular tissue which surrounds the bas fond, and the lateral portions of the bladder ; and we see inflammation of this tissue spreading to all the soft parts within the pelvis, the rectum, and the peritonæum. In other cases the neck of the bladder and the left half of the prostate are found scarcely divided by the incision, but exhibiting a wound of which the lips are contused, torn, stretched, and the surface soft and gangrenous. In this case, also, the inflammation extends into the pelvis, to the rectum, and to the peritonæum ; but from different causes.

The *bladder itself may become inflamed* by being pinched, stretched, bruised, or lacerated with the forceps. This instrument, passed between the bladder and rectum, may seize the stone between the coats of the bladder ; or the posterior wall alone of the bladder may be embraced by the instrument.

The cellular tissue of the *rectum may become inflamed* from the violence done to it ; or its mucous membrane may be inflamed by the contact of urine arising from an opening through the gut.

*Estimate of the principal Instruments employed.*—Speaking of the various instruments in common use, Dr. Stevens observes :—

" In the lateral operation, the preference of one or other of these instruments is more or less a matter of taste, about which no one should dispute. But as regards the bistouri cachê of Frère Côme, I consider it a dangerous instrument; liable to wound the fundus of the bladder, and by no means necessarily making, in the withdrawal of it, the definite incision claimed as one of its advantages. The incision must be larger or smaller, not only according to the graduation of the instrument, but according as it is withdrawn straight, or pushed to one side or the other ; or as its handle is elevated or depressed. The knife of Langenbeck, and the straight staff of Mr. Key, appear to me very awkward instruments: after one trial of each of them, I fully resolved not to employ either of them again. The blunt bistoury seems to me a very convenient instrument; but considering that the incision of the prostate is to be made with one sweep of the knife, it must happen that this incision is more or less extensive than the operator may desire, according as that gland is harder or softer, or larger or smaller. The desideratum is to make an incision of a definite extent; this object is not precisely attained by the bistoury."

Dr. Stevens refers to the observations of Mr. Stanley on the subject. They are, like all the remarks of that gentleman, judicious.

An exclusive preference is not to be given to the gorget, or to the knife for the incision of the prostate. With either instrument, skilfully used, the operation may be well done. With a gorget, properly constructed, there is no risk of wounding the internal pudic artery or the rectum, because the limits of the incision are determined by the dimension and form of the instrument. With a knife in an experienced hand, there is not so much certainty of confining the incision within its proper limits.

A comparison of the gorget with the knife, so far as instituted, is favourable to the former ; but to the narrow-bladed and beaked knife, first used by Mr. Blizard, an advantage belongs, which a gorget, from the width of its blade, cannot possess. The knife enters the bladder, as Mr. Blizard was accustomed to remark, as easily as a probe. The gorget, on the other hand, must meet resistance in passing through the prostate ; very much less, however, will this resistance be, than it has been usually represented, when the gorget has been properly made, and it is guided with skill.

For the young subject, or for a thin adult, the knife is especially suited. It is also to be preferred for any case in which the bladder is closely contracted upon the stone. But for a very fat, or for an old subject, in whom, by the enlargement of the prostate, or the dilatation of the rectum, the bladder is raised much above its natural situation, the gorget is better adapted, on account of the great distance from the perineum, at which the prostate and neck of the bladder are in such instance situated.

We must say that we think a good gorget not so bad an instrument as it is now generally represented to be. One great advantage attending it, is the obliquity of its handle, in reference to its blade. When the scalpel is introduced into the groove of the staff, a clumsy manœuvre is requisite to accommodate the operator's hand to the subsequent stage of the incision.

*Best Method of Performing the Operation.*—Dr. Stevens revives, but he believes, improves the Celsian operation. The form of incision is much the same as that of the " cutting on the gripe," but it is the staff, not the gripe that he cuts on.

He offers to the profession a new instrument for the bi-lateral section of the prostate ; in form it resembles a large olive, with a beak at the extremity, with cutting edges at the sides, parallel to its longest axis, and with a straight handle. The instrument, of which there are three sizes, and the manner of employing it, will be readily understood by the annexed engravings. The grooved staff employed in connexion with this instrument, is as wide as the urethra will admit, and the groove gradually terminates as it approaches the end of the staff. The advantages in the use of this instrument are, first, that the circular form of a transverse section, gives an opening through the gland of three diameters instead of two, as when a flat instrument is employed ; thus it is not necessary to carry the incision so far laterally to obtain an opening of given dimensions ; and hence there is less likelihood of hemorrhage from injuring the plexus of vessels that surrounds the prostate.

Second. The prostate is cut horizontally, and though not absolutely, yet for all practical purposes in its greatest diameter.

Third. The rectum is pushed back by by the convexity of the posterior part of the instrument.

Fourth. As the prostate is stretched transversely across the instrument, the section is made by a clean cut, and with so little resistance that the instrument does not, like ordinary gorgets, require to be thrust in with force, but may be passed lightly along until the section is completed. Thus there is less danger of wounding the fundus of the bladder by a sudden cessation of resistance from the parts divided ; they are, in fact, divided without force.

Fifth. The easy division of the prostate obviates the danger of tearing the cellular tissue which connects the anterior surface of the bladder to the posterior wall of the ossa pubis.

*Mode of Operating.*—" The following is the method I have adopted, and the one I would recommend for performing the operation.

Let the patient be secured in the usual manner, but with the pelvis rather higher than the trunk, on a firm flat table, with only two or three thicknesses of blanket under him. The rectum should have been previously emptied by a dose of oil given on the night preceding the operation, and the urine should be retained for a short time previous. The patient secured, let the assistant who is to hold the staff be placed on the patient's left, and a second assistant on his right, to support the rectum with a cloth, in case of prolapsus. The staff is then to be introduced. If you are quite certain of the existence of a stone, I would advise you not to delay the operation because you do not feel it when the patient is on the table ; and much less would I advise you to ask all the assistant surgeons to satisfy themselves of the presence of the calculus. During these painful and protracted manœuvres, the urine is often discharged, and you lose the advantage of its presence in the bladder. Let only one of the assistants sound, and in doing so press the urethra against the groove of the staff to prevent the escape of urine. Exhort your patient not to strain ; and while you are encouraging him with the hope of speedy relief, mark with your eye, but more especially by feeling with your finger, the exact situation of the two tuberosities of the ischia ; the divergence of these two bones, the lower border of the triangular ligament, and the bulb of the urethra. The bulb is situated directly at the lower border of the ligaments, which to pretty firm pressure will impart a greater degree of resistance than the parts between it and the anus. In a deep pelvis and with enlarged prostate, you may expect to find a deep perinæum, and should be prepared to find the bladder more than usually out of reach. Plan for yourself an incision of a crescentic shape, posterior to the bulb, but near it—nearest it when the pouch of the rectum, as in old men, is much enlarged, and in those whose pelvis is naturally narrow at the inferior strait. Let its convex side be next the bulb, its horns between the anus and the tuberosities of the ischia, not below the centre of the anus, for here are the hemorrhoidal vessels ; not within a quarter of an inch of the ischia, for by approaching the bone too closely, the incision might reach the internal pudic artery. Now, covering the anus with three fingers of the left hand, press the rectum backward and tighten the skin of the perinæum. As a general rule, make your incision one inch and a quarter anterior to the anus, and in length about one inch and three quarters, with slight convexity forwards. When the incision through the skin is completed, pass the fingers of the left hand into the wound, so as to tighten the fascia to be cut and to press back the rectum. If you fear you are approaching too near the bowel, put your finger into the rectum, and ascertain the relations of the incision to that part. Continue the incisions in the same plane, but rather more forward, especially if you find your cut very near the rectum, for the gut bulges forward as it ascends. Continue to press back its anterior walls,—now pass the finger deep into the centre of the wound, and turning forward its radial edge, feel for the staff and for the bulb of the urethra. Holding your finger upon the bulb and the nail upon the staff, pass a small bistoury beyond it, and cut the membraneous part of the urethra upon the groove of the staff. This part of the urethra is surrounded by a dense fibrous sheath, the knife for dividing it should be sharp at the point or very narrow. It is not uncommon to witness delay in exposing the staff; this I believe arises from not having the urethra stretched by a large staff, from mis-calculating the thickness of the fibrous sheath surrounding it, or lastly, from using a broad-pointed knife. The membranous portion being divided, pass the beak of the bisector into the groove, and holding the staff with the left hand, glide the bisector with the right hand along the groove until the escape of urine advises you that the section is completed. The angle which the bisector should make with the staff, should be just enough to keep the beak in the groove. If the patient is young, you may be well assured that you can reach the bladder with the finger. So you will in adults and old persons, unless the pelvis is deep or the prostate enlarged. In the latter cases only you may leave the staff in the bladder to guide the forceps; but in my own practice, I have always withdrawn the staff as soon as the section of the prostate was completed, introducing my finger into the wound at the same moment."

*Of the Resistance to the Extraction of a Calculus.*—After several remarks upon the bladder, bones of the pelvis, &c. Dr. Stevens sums up, by saying:—

"I consider the resistance to arise from the capsule of the prostate, and the neck of the bladder; from the transversalis muscles when not divided on either side; from the levator ani and its investing fasciæ."

The following experiment, which was made upon a male subject, about 35 years of age, four days after his death; in cold weather, is instructive:—

"A grooved staff was introduced into the bladder, and a transverse incision, one inch and a half long, and one inch anterior to the anus, was made upon the staff into the membraneous part of the urethra, and the prostatic bisector was introduced along the groove into the bladder. The bisector and staff, placed in position, measured three inches and a half in circumference. The fundus of the bladder being then opened, it was ascertained that the incision had approached within one or two lines of the sides of the prostate, which, however, were not entirely divided.

A large forceps was then introduced, and an egg-shaped stone properly placed within its grasp. The stone and forceps, measured together, gave a circumference of nearly six inches. The instrument, with its blades perpendicular, was then pulled upon with a force that might have been sufficient to lift a weight of twenty pounds, and moved from side to side and up and down. The largest circumference of the stone did not pass out of the bladder until the traction was increased to forty pounds. A very moderate degree of force then sufficed for its removal; but there was a sense of tearing, as the stone passed through the external incisions. It seemed that the fibrous capsule of the prostate had offered the first and greatest resistance; the levator ani the last.

The tract of the wound was next exposed to view, by extending the left side of the incision through the bones and soft parts, so as fairly to lift them over to the right side of the pubes.

In the posterior plane was the lower half of the prostate apparently not much contused or lacerated, (the lower blade of the forceps had covered this;) between it and the anus were the longitudinal fibres of the rectum about a line in thickness. On the right side of the prostate were some stretched and lacerated fibrous shreds, like fine cotton thread, connecting the upper and lower halves of the prostate at their edges. Between this part and the right extremity of the external incision, were indistinct portions of muscular fibres, parts of the levator ani and of the transversalis. On the left side of the prostate, where the incision had been extended after the removal of the stone, a large plexus of vessels, filled with venous blood, was brought into view. The parts were then carefully smoothed with the handle of the scalpel, and with this and the finger the loose cellular tissue was separated until three pouches or cavities, more or less connected with the tract of the wound, were brought into view;—the first, high up on the right side, was bounded externally by the obturator internus muscle, internally by the bladder and prostate, and inferiorly by the levator ani; below the levator ani was another pouch which extended in front of the sacrum and behind the rectum; between the rectum and the bladder was a third cellular space in which the cellular structure was rather less loose than in the other two."

The Lecture from which we have made the preceding extracts, will repay perusal amply.

---

### Royal Infirmary of Edinburgh.

The Dangers of Lithotrity. By W. Fergusson, F.R.C.S. E. &c., one of the Surgeons to the Royal Infirmary of Edinburgh.*

Our readers are aware that, from the first, we pointed out the exaggerated notions that were entertained by many and expressed by some, in reference to the value of lithotrity. Time has in all respects confirmed our anticipations, and the danger at present appears to be an unreasonable dislike to the operation. Lithotrity has led to the crushing and extraction of small stones—to the better sounding of the bladder—to the investigation of the symptoms of calculus at an early stage by surgeons, and to a correspondingly early application for assistance by patients. Doing this, lithotrity has done immense service to humanity.

Dr. Fergusson makes some very sensible remarks upon lithotrity. It is not our purpose to quote these, but simply to lay before our readers the facts that have fallen under his own observation.

"I shall state briefly the particulars of certain cases which have occurred in my own practice, and the results of a considerable number more, where I have had ample opportunities of making myself fully acquainted with the details."

The facts then are the following.

*Case* 1.—A stout healthy man, about the middle period of life, underwent repeated operations with the lithotrite, all of which were attended with great pain; and though at the end of several years no stone could be felt, he was in a worse condition than before coming under the surgeon's hands, in consequence of chronic disease of the bladder, apparently induced by the method of treatment.

*Case* 2.—Mr. J. was operated on several times with very little suffering. After being fourteen days under treatment, only one small fragment could be detected, which, however, could not be readily grasped with the instrument; he went to the country, and shortly after passed a fragment, supposed to be the remainder of the stone. Not long after, symptoms of stone again came on, and about eighteen months after the first operation, he submitted again to lithotrity, and the operation was performed with apparent success.

* Edinburgh Med. and Surg. Journal, Oct. 1838.

*Case* 3.—J. W. submitted to lithotrity, and bore the first operation without much pain. In the subsequent attempts, he suffered torment, but, notwithstanding, determined to persist, though it was frequently proposed to perform lithotomy. After repeated operations, and great suffering, the bladder seemed clear of all fragments; and ever since, he has remained in good health.

*Case* 4.—R. B. had suffered from stone in the bladder for forty years, firmly resolved never to submit to lithotomy. He felt anxious to undergo lithotrity, and put himself under my care for that purpose. I made an attempt to seize the stone without success, and though the smallest possible degree of violence was used, still he suffered excruciating pain, which was followed by a feverish attack, that did not leave him for several months, and since then he remained in a very exhausted condition. After his health was somewhat restored, he at last submitted to the operation of lithotomy, and I removed a large mulberry stone, weighing nearly five ounces. He made a rapid recovery.

*Case* 5.—The Rev. M. A. subjected himself to lithotrity, but suffered so much after the first attempt, that it was not considered advisable to proceed. Twelve months afterwards, he put himself under my charge, and I removed a small stone by the lateral operation. He made a slow recovery, but, like the patient whose case is last referred to, declared, that in the event of being again the subject of this disease, he would rather submit a second time to lithotomy than to lithotrity.

*Case* 6.—M. M. much against my advice, submitted to lithotrity; the operation, though performed with the least possible violence, was followed with excessive irritation and pain in the bladder, which continued for the next twelve hours, but was subdued by powerful opiates. In thirty-six hours, the pain returned, and continued without intermission until death, which happened four days after the operation was done. On dissection, the fragments of a large stone were found in the bladder, and a small one untouched. The prostate gland was enlarged, more particularly the middle lobe. The bladder was sacculated, but there was no trace of any injury having been inflicted on it during the operation. Both kidneys were much diseased in this case, and the psoas muscle on the left side seemed converted into a soft mass resembling coagulated blood.

*Case* 7.—Mr. C. was severely afflicted with symptoms of stone. He consulted me as to the propriety of lithotrity, but I strongly dissuaded him from it. Shortly afterwards, however, he put himself under the care of a professed lithotritist, who broke the stone on one occasion, but could never induce him to submit to a second operation. He has since then suffered much, and is now in a state of great misery, being no doubt in a more deplorable state than before the stone was broken into fragments.

"Many other cases," he proceeds, "have come under my own knowledge or observation with which, however, I was not so particularly connected as those referred to above. I shall, therefore, only mention them in the following list, which will show the result of my experience and knowledge in cases of lithotrity, which have not been described in any accounts on the subject, as yet published.

Out of eighteen cases in which lithotrity was performed, six have been cured; seven not cured, and five have died.

In one of the number of cured, there are strong reasons to suspect a return of the disease; in another, though no stone can be felt, the patient has suffered almost as much since he was operated on (in consequence of disease of the bladder induced thereby,) as he did previous to coming under the surgeon's care. Indeed, in two of these cases only can the operation be said to have been attended with that happy success, which has been so generally claimed for lithotrity.

Of the seven not cured, four afterwards underwent lithotomy, of whom one died and three recovered.

